# Principal component analysis for three-dimensional structured illumination microscopy (PCA-3DSIM)

**DOI:** 10.1038/s41377-025-01979-8

**Published:** 2025-09-01

**Authors:** Jiaming Qian, Weiyi Xia, Yuxia Huang, Jing Feng, Qian Chen, Chao Zuo

**Affiliations:** 1 Smart Computational Imaging (SCI) Laboratory, Nanjing University of Science and Technology, Nanjing, Jiangsu Province 210094 China; 2 Smart Computational Imaging Research Institute (SCIRI) of Nanjing University of Science and Technology, Nanjing, Jiangsu Province 210094 China; 3 Jiangsu Key Laboratory of Spectral Imaging & Intelligent Sense, Nanjing University of Science and Technology, Nanjing, Jiangsu Province 210094 China

**Keywords:** Microscopy, Imaging and sensing

## Abstract

Three-dimensional structured illumination microscopy (3DSIM) is an essential super-resolution imaging technique for visualizing volumetric subcellular structures at the nanoscale, capable of doubling both lateral and axial resolution beyond the diffraction limit. However, high-quality 3DSIM reconstruction is often hindered by uncertainties in experimental parameters, such as optical aberrations and fluorescence density heterogeneity. Here, we present PCA-3DSIM, a novel 3DSIM reconstruction framework that extends principal component analysis (PCA) from two-dimensional (2D) to three-dimensional (3D) super-resolution microscopy. To further compensate spatial nonuniformities of illumination parameters, PCA-3DSIM can be implemented in an adaptive tiled-block manner. By segmenting raw volumetric data into localized subsets, PCA-3DSIM enables accurate parameter estimation and effective interference rejection for high-fidelity, artifact-free 3D super-resolution reconstruction, with the inherent efficiency of PCA supporting the tiled reconstruction with limited computational burden. Experimental results demonstrate that PCA-3DSIM provides reliable reconstruction performance and improved robustness across diverse imaging scenarios, from custom-built platforms to commercial systems. These results establish PCA-3DSIM as a flexible and practical tool for super-resolved volumetric imaging of subcellular structures, with broad potential applications in biomedical research.

**Figure:**
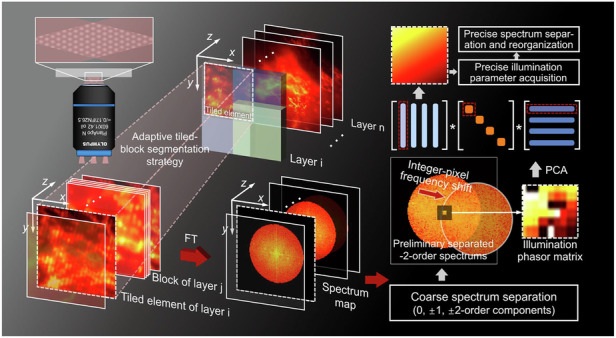
This article developed PCA-3DSIM, a mathematically grounded enhancement to 3D structured illumination microscopy that improves robustness by integrating physical modeling with statistical analysis.

This article developed PCA-3DSIM, a mathematically grounded enhancement to 3D structured illumination microscopy that improves robustness by integrating physical modeling with statistical analysis.

## Introduction

Visualizing three-dimensional (3D) structures of cells and subcellular components at the nanoscale is crucial in the fields of life sciences and biomedicine^1–4^. Among various 3D super-resolution imaging techniques, 3D structured illumination microscopy (3DSIM) stands out thanks to its advantages of excellent optical sectioning, low illumination dose, fast acquisition speed and compatibility with regular fluorophores, and has been widely applied to observe volumetric nanoscale organizations and functions of live cells^5–12^.

3DSIM excites the sample using structured illuminations with sinusoidally distributed intensities, which encode high-frequency signals beyond the diffraction limit into the system’s passband, thereby enabling a significant improvement in both lateral and axial resolution-typically by up to a factor of two^13,14^. The recovery of super-resolution information critically depends on numerical reconstruction algorithms, where the extraction of illumination parameters plays a key role in accurately decoupling modulated signals and optimizing user-defined parameters to suppress artifacts during image reconstruction^15,16^. In existing solutions, both commercial and open-source, the experimental illumination parameters (e.g., wave vector, initial phase, modulation depth)—assumed to be constant across the full field-of-view (FOV)—are typically calculated based on a single slice with the optimal modulation from the 3D stack, or a composite slice synthesized from the 3D stack, and subsequently applied to reconstruct the entire dataset^17–19^. However, uncertainties such as optical aberrations, mechanical errors, fluorescence density heterogeneity, environmental disturbances, dysregulated modulation depths and other experimental imperfections can cause fluctuations in the actual illumination parameters across the volumetric space^20^. Therefore, breaking the assumption of constant illumination parameters in the case of substituting a slice for volume will inevitably lead to reconstruction artifacts that compromise fidelity and quantification. The current primary attention is paid to eliminating the artifacts already available in the super-resolved images^21–25^. However, some reconstruction errors are difficult to correct unless they are accounted for at the source, such as frequency misalignments due to inaccurate wave vectors, which becomes more complex when optimal illumination parameters are spatially perturbed. Such limitations have been proven to be alleviated by a tiled reconstruction strategy that improves the reconstruction quality of 3DSIM data^26^. However, this strategy accounts only for lateral variations in illumination parameters, which limits its effectiveness when imaging thicker specimens. For instance, aberrations caused by thick samples can distort the illumination patterns, degrading pattern visibility and final image contrast^27–30^. Similar approaches have also been extended to optical sectioning imaging of thick specimens^31,32^. However, these works focus on enabling high-throughput imaging across large areas beyond the FOV constraints of high-magnification objectives, primarily to address stitching-related artifacts. In contrast, applying tiled reconstruction within a volumetric dataset confined to a single FOV remains challenging, as local subregions often exhibit reduced informative content and lower signal-to-noise ratios (SNRs), which can result in less reliable illumination parameter estimation compared to those derived from the full-FOV single-layer data.

Recent advancements in computational methods have sought to mitigate these issues. One such approach is principal component analysis (PCA), which has been successfully applied in 2D structured illumination microscopy (PCA-SIM)^33^. However, extending PCA to 3DSIM introduces additional complexities related to the higher data dimensionality and the varying illumination parameters across the sample volume. In this work, we introduce PCA-3DSIM, an extension of PCA to 3D super-resolution microscopy. Unlike the traditional slice-for-volume method, PCA-3DSIM determines precise illumination parameters in the 3D space with high efficiency, while effectively filtering out illumination-irrelevant interferences. Additionally, an adaptive tiled-block strategy is integrated to address spatial nonuniformities in the illumination parameters by segmenting the raw 3D stack into volumetric tiled-block subsets of various types oriented to the reconstruction quality, with each subset individually parameterized to ensure high-fidelity, artifact-free 3D super-resolution reconstruction. We experimentally validate the effectiveness and robustness of PCA-3DSIM on both self-developed and commercial 3DSIM devices, demonstrating its capability to provide reliable and high-quality super-resolution reconstruction.

## Results

### Principle of PCA-3DSIM

#### 3DSIM reconstruction based on high-order principal component analysis

**Fig. 1:**
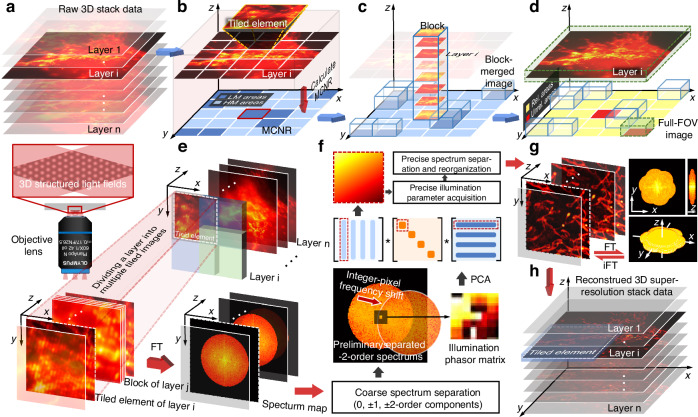
**Flow chart of adaptive tiled-block 3D structured illumination microscopy based on principal component analysis**. **a**–**d** Schematic diagram of the adaptive tiled-block reconstruction strategy, where **a** is the raw 3D stack data, **b** represents the tiled images segmented from the full-FOV image of the *i*-th layer, and **c**, **d** illustrate the block merging operation based on MCNR for tiled images with low modulation and the operation of replacing tiles with whole layers for regions with unreliable parameter estimates. **e**–**h** Algorithmic steps for 3D super-resolution reconstruction based on PCA-3DSIM: Step 1: perform segmentation of different types on the raw 3D stack based on the adaptive tile-block strategy, and obtain the spectral information of each subset (**e**); Step 2: for each subset, remove the parameter-independent interference based on PCA and obtain high-quality illumination parameters (**f**); Step 3: reconstruct each subset based on illumination parameters (**g**); Step 4: use the reconstructed subsets into the full-FOV 3D super-resolution stack information (**h**)

In 3DSIM, the emitted sample fluorescence modulated by structured illumination is captured layer-wise along the axial direction, resulting in a series of raw 3D stack data, as shown in Fig. 1a. For the illumination image of any given layer, its Fourier spectrum contains seven overlapping components, i.e., one 0-order frequency component representing the diffraction-limited wide-field information [${C}_{0}={a}_{0}{e}^{j{\varphi }_{0}\cdot 0}\,{\tilde{S}}_{0}({k}_{x,y},{k}_{z})\,O({k}_{x,y},{k}_{z})$], a pair of $\pm$1-order components extending the axial support domain [${C}_{\!\!\pm 1}={a}_{1}{e}^{\mp j{\varphi }_{0}}{\tilde{S}}_{\pm 1}({k}_{x,y}\pm {p}_{x,y},{k}_{z})\,\frac{1}{2}[O({k}_{x,y},{k}_{z}-{p}_{z})+O({k}_{x,y},{k}_{z}+{p}_{z})]$], and a pair of $\pm$2-order components contributing to lateral super-resolution [${C}_{\!\!\pm 2}={a}_{2}{e}^{\mp 2j{\varphi }_{0}}{\tilde{S}}_{\!\!\pm 2}({k}_{x,y}\pm 2{p}_{x,y},{k}_{z})O({k}_{x,y},{k}_{z})$] (where $k$ represents the spatial frequency coordinates, $S$ is the desired object function, the superscript $\sim$ denotes the Fourier transform of the corresponding variable, $O$ represents the optical transfer function (OTF), ${p}_{x,y}/{p}_{z}$ is the lateral/axial wave vector, ${\varphi }_{0}$ represents the initial phase, and ${a}_{n}$ denotes the modulation depth of the n-order frequency components). More details about the information modulation in 3DSIM are provided in Supplementary file 1: Note S1 and Supplementary file 2: Movie 1. To recover high-quality super-resolution images, precise acquisition of experimental illumination parameters (${p}_{x,y}/{p}_{z},{\varphi }_{0}$ and ${a}_{n}$) is necessary for decoupling and reassembling the originally overlapping frequency components (${C}_{0},{C}_{\!\!\pm 1}$ and ${C}_{\!\!\pm 2}$).

Unlike the conventional approach of substituting a slice for volume (see Supplementary file 1: Note S2 for details), we are dedicated to retrieving the illumination parameters in a 3D manner from the stack data. Taking the -2-order spectrum component as an example, after ignoring the influence of the system OTF and the modulation depth, its inverse Fourier transform after an initial frequency shift can be expressed as $S({r}_{x,y},z){e}^{j({p}_{sub}\cdot {r}_{x,y}+2{\varphi }_{0})}$ (where ${r}_{x,y}$ and $z$ denote the spatial coordinates in the lateral and axial directions, respectively, and ${p}_{sub}$ is the sub-pixel portion of the wave vector $2{p}_{x,y}$). With respect to the phasor term ${e}^{j[{p}_{sub}({r}_{x,y},z)+2{\varphi }_{0}]}$, which can be characterized as a 3-order tensor ${\mathcal I} \in {R}^{{N}_{x}\times {N}_{y}\times {N}_{z}}$ (where ${N}_{n}$ represents the data length on the n-axis), it is not difficult to see that the slope and constant term of its phase expression are the numerical reflections of the sub-pixel wave vector ${p}_{sub}$ and the initial phase${\varphi }_{0}$, respectively. However, the readily accessible ${e}^{i\cdot angle\{{ {\mathcal F} }^{-1}[{C}_{-2}(k+{p}_{{\rm{int}}})]\}}$ differs significantly from its ideal form ${\mathcal I}$, with noise, OTF attenuation, and other interferences hindering the precise extraction of illumination parameters (where $angle$ denotes the function that returns the phase, ${ {\mathcal F} }^{-1}$ represents the inverse Fourier transform operation, and ${p}_{int}$ is the integer-pixel portion of the wave vector, which can be obtained by simple peak localization). From the perspective of data features, any lateral and axial slice of tensor ${\mathcal I}$ can be decomposed into the product of two orthogonal vectors according to the exponent addition property, implying that the Tucker-rank of ${\mathcal I}$ in any mode is one, i.e., matrices unfolded from ${\mathcal I}$ along different modes are essentially rank-one^34^. Based on the above analysis, the ideal phasor tensor can be attained from the first principal component extracted by high-order singular value decomposition (HOSVD)^35,36^ of ${e}^{i\cdot angle\{{F}^{-1}[{C}_{-2}(k+{p}_{{\rm{int}}})]\}}$. Taking the x-mode unfolding matrix ${ {\mathcal I} }_{(x)}\in {R}^{{N}_{x}\times ({N}_{y}\cdot {N}_{z})}$ as an example, it can be further expressed as:

1 $${ {\mathcal I} }_{(x)}={s}_{x}{s}_{y,z}^{H}$$

where ${s}_{x}={e}^{j({p}_{x,sub}\cdot {r}_{x}+2{\varphi }_{x,0})}$, ${p}_{x,sub}$ and ${\varphi }_{x,0}$ are the x-direction components of the wave vector and the initial phase, ${s}_{y,z}$ is a vector of similar form unfolded along the z-direction, and ${\{\cdot \}}^{H}$ represents the complex conjugate transpose operations of the original object respectively. Since the unfolding matrix is rank-one, ${s}_{x}$ can be directly characterized by the singular vector corresponding to the first principal component in ${ {\mathcal I} }_{(x)}$, meaning that ${p}_{x,sub}$ and ${\varphi }_{x,0}$ are conveniently available. Therefore, by simply performing linear regression on the principal singular vectors of n-mode unfolding matrix, the precise illumination parameters (${p}_{sub}$ and ${\varphi }_{0}$), with noise and other high-dimensional data components filtered out, can be acquired. More algorithm details are provided in Supplementary file 1: Note S3.1.

Regarding the axial illumination parameter ${p}_{z}$, the main effect of which is the axial expansion of OTF, it can be accessed according to the angular relationship between the three amplitude wave vectors involved in the interference:

2 $$\frac{{p}_{x,y}}{\sin \beta }=\frac{{p}_{z}}{1-\,\cos \beta }$$

where $\beta$ denotes the angle between the center beam and the two side beams. The above procedure can be regarded as an extension of the PCA-SIM framework^33,37^ we previously developed in 3DSIM. Subsequently, spectral reorganization and reconstruction can be performed to obtain super-resolved information. The entire algorithm flow is provided in Supplementary file 3: Movie 2.

#### Adaptive tiled-block 3DSIM reconstruction based on principal component analysis

##### Adaptive tiled-block 3DSIM reconstruction

Although illumination parameters can be measured from stack data using high-order PCA, only one set of parameters is obtained, which, like the slice-as-volume approaches^17–19^, is inherently incapable of addressing the issue of optimal parameter solutions varying with volume. To further cope with non-uniformity of parameters across the volume due to factors such as aberration distortion caused by thick samples^27–30^, a tiled reconstruction strategy^26^ is adopted to accommodate spatially-variable parameters.

First, as shown in Fig. 1a, b, the raw 3D stack data, with side lengths of $N$ pixels and ${N}_{z}$ layers, is divided into equally sized sub-stacks, with each layer of the sub-stack referred to as a tiled element/image:

3 $${D}_{i,j}(x,y,z)=D[m(i-1)+1:m(i+1),\,m(j-1)+1:m(j+1),\,z]$$

where ${D}_{i,j}$ represents the subset of row $i$ and column $j$ (total ${(\frac{2N}{m}-1)}^{2}$ sub-stacks with size $2m\times 2m\times {N}_{z}$), and $D$ denotes the captured raw 3D stack. The determination of the sub-stack size will be explained in Supplementary file 1: Note S4. Each ${D}_{i,j}$ can be reconstructed with independently measured illumination parameters and user-defined parameters based on these measurements, thus alleviating reconstruction artifacts caused by lateral distortions^26,38^. Ideally, illumination parameters of the same orientation and phase shift keep consistent along the axial direction, which do not vary with the position of the sample. However, mechanical scanning, volumetric fluorescence variations and environmental disturbances can induce axial interference. As a result, individual parameter extraction needs to be performed for each tiled element in the sub-stack. Taking into account that some tiled elements may be cropped from background or out-of-focus scenes, making it difficult to provide precise parameters, a modulation-guided criterion is utilized to further filter elements potentially contributing reliable values. The modulation depth of the illumination patterns in 3DSIM can be characterized by the modulation contrast-to-noise ratio (MCNR), which is also a measurement of the illumination quality^39,40^. To calculate MCNR, the photon counts for each pixel are obtained by performing a one-dimensional (1D) Fourier transform on each pixel in the tiled element along the sequence of the five-step phase-shifting images:

4 $$N(n)={A}_{0}+{A}_{1}\,\cos \left(\frac{2\pi n}{5}+{\alpha }_{1}\right)+{A}_{2}\,\cos \left(\frac{4\pi n}{5}+{\alpha }_{2}\right)$$

where $n$ $(n=1,2,3,4,5)$ represents the number of phase shifts, and ${A}_{u}$ and ${\alpha }_{u}$ denote the u-order Fourier coefficients and the u-order Fourier phases, respectively. The modulation depth in MCNR is taken as twice the mean value of the square root of the 1- and 2-order Fourier coefficients (${A}_{1}$ and ${A}_{2}$), and the shot noise level is the square root of the 0-order Fourier coefficient (${A}_{0}$). Thus, MCNR for each pixel can be written as:

5 $$MCNR=\frac{2\sqrt{{A}_{1}^{2}+{A}_{2}^{2}}}{\sqrt{{A}_{0}^{2}}}$$

The above calculation itself is a form of SIM reconstruction, where the 2-order Fourier coefficients generalize the conventional optical sectioning scheme, typically achieving satisfactory results only within a limited range of focal positions. Therefore, as illustrated in Fig. 1b, c, we conduct layer-independent parameter calculations for tiled images with MCNRs reaching a specific threshold, while the others are replaced with composite focal slices^19^ (obtained by weighted merging of pre-filtered elements within a sub-stack based on MCNR, i.e., block merging operation), thereby reconciling the conflict between axial distortion and parameter extraction accuracy:

6 $${D}_{i,j}^{{\prime} }({r}_{x,y},z)=\left\{\begin{array}{l}{D}_{i,j}({r}_{x,y},z),MCN{R}_{{\rm{norm}}}(z) > {M}_{t}\\ \mathop{\sum }\limits_{z=1}^{{N}_{z}}{D}_{i,j}({r}_{x,y},z)\frac{MCN{R}_{{\rm{norm}}}(z)}{MCN{R}_{{\rm{norm}}}(z)},else\end{array}\right.$$

where $D^{\prime}$ represents the tiled image used for parameter estimation, $MCN{R}_{norm}$ denotes the normalized MCNR, and ${M}_{t}$ is the threshold for judging modulation quality, which is empirically set to 0.85. It should be noted that the use of composite focal slices, rather than lateral images, is driven by their ability to mitigate the impact of axial errors through superposition, while the tile segmentation effectively addresses lateral aberrations. Meanwhile, to avoid issues of parameter estimation anomalies that may still occur with some of the block-merged tiled data, we apply experimental illumination priors to directly evaluate the quality of the acquired parameters. In simple terms, the standard deviations of the lateral wave vectors ($S{D}_{wv}$) and angles ($S{D}_{ang}$) for illumination parameters at three orientations are calculated. Based on the prior that the lateral wave vectors from different orientations are numerically close and exhibit an angular difference of 120°, any parameters with $S{D}_{wv}$ or $S{D}_{ang}$ exceeding a specific threshold ($S{D}_{wv}$> 0.5 or $S{D}_{ang}$> 5) are considered anomalous and will subsequently be replaced by those from the layer where the tiles are located (Fig. 1d). Based on the above strategy as shown in Fig. 1a–d, the raw 3D stack data can be adaptively segmented into a series of volumetric tiles, blocks, or layers capable of being processed independently oriented by the reconstruction quality, thus dealing with the issue of local perturbation in the optimal reconstruction parameters due to complicated 3D distortions caused by various uncertainties. Under the adaptive tiled-block strategy, the raw data have been segmented into a series of tiled images distributed along lateral and axial directions in volume space, so that regular PCA, which is more efficient and convenient compared to high-order PCA, can be directly employed to derive 3D illumination parameter distributions.

In the Fourier domain of each tiled image (Fig. 1e), the initial separation of the individual overlapping components can be carried out through known phase shifts, followed by integer-pixel spectral shifts based on the localized frequency peaks. Referring to section “3DSIM reconstruction based on high-order principal component analysis”, the illumination phasor term obtained from the -2-order spectrum components containing the sub-pixel wave vector and the initial phase can be expressed as ${e}^{j[{p}_{sub}\cdot {r}_{x,y}+2{\varphi }_{0}]}$. Based on the fact that the ideal illumination phasor matrix is essentially rank-one^41^, which contains only a single principal component, SVD is performed to search for the first principal component of ${e}^{i\cdot angle\{{ {\mathcal F} }^{-1}[{C}_{-2}(k+{p}_{{\rm{int}}})]\}}$ dominated by the illumination parameters:

7 $${e}^{j[{p}_{sub}\cdot {r}_{x,y}+2{\varphi }_{0}]}\approx U\varLambda {V}^{T}$$

where the superscript $T$ denotes the transpose operation on the matrices, $\varLambda$ is the semi-positive definite diagonal matrix whose diagonal retains only the same number of eigenvalues as the rank, and $U$ and $V$ are the unitary matrices consisting of the left singular vectors and the right singular vectors, respectively. By calculating the slopes of the 1D unwrapped phase distributions of the left and right dominant singular vectors in Eq. 4, the residual sub-pixel wave vector ${p}_{sub}$ can be determined, while filtering out high-dimensional interference terms in the phasor matrix that are unrelated to the illumination parameters. The phase of the illumination phasor matrix at point 0 can also be extracted, which is actually the initial phase. However, PCA itself is still time-consuming and its results are vulnerable to weak signals. Since the inverse Fourier transform of the ideal illumination phasor is a downsampled 2D Dirichlet function with main energy concentrating in limited support surrounded by noise distributions, we apply a frequency-domain masking operator to retain the energy-concentrated spectral peak region and zero out the region with low SNRs. The utilization of the masking operator substantially enhances SNRs while decreasing the data amount, resulting in significant improvement in both accuracy and speed of the subsequent parameter computation procedures compared to the conventional iterative cross-correlation method^6^. The complete flowchart of PCA-based illumination parameter estimation is summarized in Fig. 1f. Detailed algorithmic details are provided in Supplementary file 1: Note S3.2.

Based on the lateral illumination parameters obtained by PCA, precise separation and shifting of different frequency components can be achieved. The axially extended OTF used for deconvolution can be derived from the axial wave vector component of the $\pm$1-order spectrums obtained by Eq. 6. Consequently, each subset can be independently reconstructed to obtain 3D super-resolved volumetric data with twice the lateral and axial resolution beyond its classical limits (detailed in Supplementary file 1: Note S3.3), as illustrated in Fig. 1g.

##### Full-FOV reconstruction stack acquisition based on overlapping subset fusion

Since the raw full-FOV acquisitions are divided into a series of sub-stacks, image fusion is required to synthesize the final super-resolution images. Considering the differences in dynamic range between the subset images, when carrying out the tile segmentation of Eq. 1, a certain overlapping region between two neighboring subsets is necessary for eliminating the boundaries during image fusion, as illustrated in Fig. 1e. Regarding the selection of subset size, smaller edge length can ensure less parameter variation within a single tiled element, implying more accurate parameter acquisition. However, an excessively small size determines a limited sampling number, leading to reconstruction quality degradation instead and introducing serious computational burdens. Taking into account both parameter extraction accuracy and data processing efficiency, the size of a single tiled image is determined to be 128$\times$128 pixels, with half of its pixels overlapping with its neighboring image (see Supplementary file 1: Note S4 for more details). Given the still large data volume, we employ a simple and effective weighted averaging fusion algorithm^42^, which can enhance the overall image quality by performing weighted averaging of intensity information in the overlapping regions between two adjacent tiled images:

8 $$F({x}_{f},{y}_{f},z)=(1-\omega ){I}_{1}({x}_{f},{y}_{f},z)+\omega {I}_{2}({x}_{f},{y}_{f},z)$$

where $F$ represents the fused image, ${I}_{1}$ and ${I}_{2}$ denote the reconstructed images of two neighboring tiled elements, the subscript $f$ is used to refer to the spatial coordinates in the overlapping region, and $\omega$ denotes the weight calculated from the s-curve fitting^43^. Eventually, reconstructions of all subsets can be stitch-fused to yield optimized full-FOV 3D super-resolution stack information (Fig. 1h). The algorithm flow of PCA-3DSIM with adaptive tiled-block strategy is summarized in Supplementary file 4: Movie 3.

### Simulations to demonstrate the high reconstruction quality of PCA-3DSIM in complex scenarios with optimal reconstruction parameters perturbed spatially

**Fig. 2:**
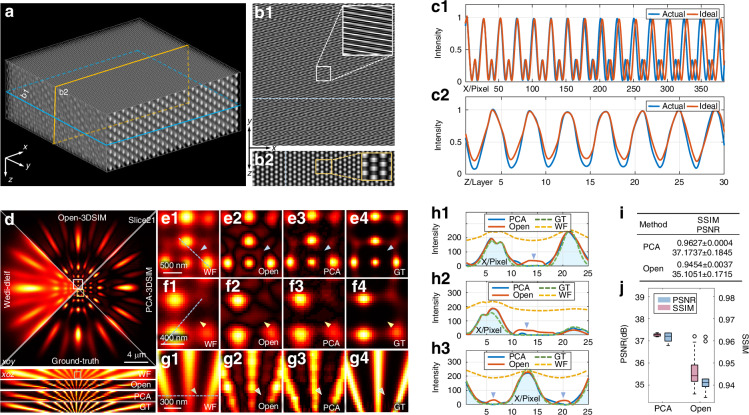
**Comparative simulation results of different methods in the case of illumination parameters varying with volume space**. **a** Simulated 3D structured illumination field with parameters varying spatially without system OTF attenuation. **b** Sliced images of the illumination field in the lateral and axial directions corresponding to the blue- and yellow-boxed regions in (**a**). **c** Intensity profiles along the blue dashed lines in (**b**), where (**c1**) and (**c2**) correspond to (**b1**) and (**b2**), respectively. **d** The wide-field image and the super-resolution images obtained by different methods (Open-3DSIM^19^, PCA-3DSIM and ground truth), where the top are lateral slices and the bottom are axial slices. **e**–**g** Magnified wide-field image and super-resolution images from the white-, yellow-, and green-boxed regions in (**d**) obtained by different methods. **h** Intensity profiles along the blue lines in (**e**–**g**). **i**, **j** SSIM and PSNR between the super-resolved images obtained by different methods and the ground truth with the addition of Gaussian noise with 5 dBW power. The simulations were independently repeated 10 times with similar results. Colored arrows point to regions where reconstruction differences are distinct. Scale bars: 4 um (**d**); 500 nm (**e**); 400 nm (**f**); 300 nm (**g**). Scale on z-axis: 30 layers, 0.125 μm per layer

We perform a series of simulations to verify the effectiveness of PCA-3DSIM. A 3D structured illumination field matching a 100$\times$, 1.4NA objective lens as shown in Fig. 2a was generated for modulation of the simulated regular volumetric structure. In order to simulate 3D distortions during the imaging process, pixel-wise wave-vector errors ranging from −1 to 1 pixel were added along a particular direction, and displacement errors of −3 to 3 nm were introduced for each layer of the modulated image stack in axial order. Figure 2b, c illustrates the intensity distributions of lateral and axial slices in the distorted illumination field, from which it can be seen that the illumination parameters are no longer constant across the full field but instead exhibit spatial disturbances. After being attenuated by the simulated 3D OTF, raw 3D stack data with the size of 384$\times$384$\times$30 was generated.

PCA-3DSIM and Open-3DSIM^19^, a state-of-the-art 3D SIM reconstruction framework, were applied to reconstruct the super-resolution images. Note that PCA-3DSIM referred to below defaults to the version with the adaptive tiled-block strategy. The super-resolved images obtained by the different methods are shown in Fig. 2d. It can be seen that Open-3DSIM attains resolution-enhanced results owing to the well-designed filtering operations against various imaging artifacts. However, its reconstruction quality is still compromised since the monolayerized measurement of illumination parameters is not applicable in the presence of 3D distortions, with several artifacts appearing in the super-resolution results, as shown in the three magnified regions of interest (Fig. 2e–g). In contrast, PCA-3DSIM, by incorporating an adaptive tiled-block operation, provides an alternative strategy that accounts for spatial variations in reconstruction parameters, helping to mitigate some of these distortions. Fluorescence intensity analysis of the super-resolution images obtained by different methods indicates that PCA-3DSIM achieves reconstruction quality comparable to the ground truth while well removing the artifacts pointed out by the blue arrows (Fig. 2h). In order to quantify the reconstruction fidelity of the two methods, we computed the structural similarity coefficient measure (SSIM) and the peak signal-to-noise ratio (PSNR) between the reconstruction results obtained by different methods and the ground truth under random Gaussian noise conditions with a power of 5 dBW, which further demonstrates that PCA-3DSIM is capable of performing more robust and higher-fidelity super-resolution reconstruction (Fig. 2i, j). Supplementary file 1: Fig. S4 provides more simulation results, including the comparison between PCA-3DSIM versions with and without the adaptive tiled-block strategy. All of these simulations indicate that PCA-3DSIM effectively addresses the issue of optimal reconstruction parameters varying across space, achieving high-quality reconstruction under complex experimental conditions.

### Experiments to demonstrate the superior reconstruction quality of PCA-3DSIM over the state-of-the-art 3DSIM reconstruction framework on data acquired by the self-developed setup

**Fig. 3:**
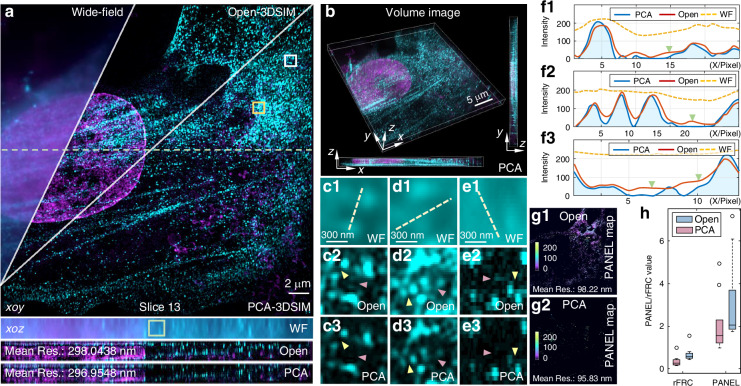
**Comparison of the multi-color super-resolution experimental results on a fixed HeLa cell sample with DAPI-labeled nucleus and FITC-labeled** ${\boldsymbol{\alpha }}$-**SMA**. **a** Comparison of the wide-field image and the super-resolution images obtained by different methods (Open-3DSIM^19^ and PCA-3DSIM), where the top are lateral slices of layer 13 and the bottom are axial slices along the green dashed line. The raw SIM images were captured through a 100$\times$ objective (UPlanXApo 100$\times$/1.45 Oil, Olympus, Japan). For easy distinguishing, we display the nucleus and $\alpha$-SMA of HeLa cells in purple and cyan, respectively. The $\alpha$-SMA is excited at 488 nm wavelength, and the nucleus at 405 nm wavelength. **b** The whole 3D sight of the super-resolution results obtained by PCA-3DSIM. **c**–**e** Magnified wide-field image and super-resolution images from the white-, yellow-, and green-boxed regions in (**a**) obtained by different methods. **f** Intensity profiles along the yellow lines in (**c**)–(**e**). **g** PANEL maps of super-resolved images obtained by different methods for a certain layer. **h** Distributions of PANEL and rFRC values for 3D super-resolution stack images obtained by different methods. The experiments were independently repeated 10 times with similar results. Colored arrows point to regions where reconstruction differences are distinct. Scale bars: 2 μm (**a**); 5 μm (**b**); 300 nm (**c**–**e**). Scale on z-axis: 16 layers, 0.125 μm per layer

We construct a 3DSIM setup based on a commercial inverted fluorescence microscope and a programmable nano-axial scanning stage (Supplementary file 1: Fig. S5) to validate the effectiveness of PCA-3DSIM in practical imaging experiments. A fixed sample of HeLa cells, with nucleus labeled by DAPI and alpha smooth muscle actins ($\alpha$-SMAs) labeled by fluorescein Isothiocyanate (FITC), was scanned and imaged along the z-axis by our customized device under the structured illumination field generated by 3-beam interference. The captured raw 3D stacks were then reconstructed using Open-3DSIM^19^ and PCA-3DSIM, respectively, with the resulting super-resolution images shown in Fig. 3. Due to inevitable instabilities in self-developed setup, maintaining consistent illumination parameters across the volume is challenging. Moreover, additional interference from factors such as optical aberrations further complicates the situation. These issues compromise the reconstruction quality of Open-3DSIM, leading not only to resolution degradation in the regions indicated by the yellow arrows, but also to the appearance of artifacts affecting the optical sectioning performance in the purple-arrowed regions, as illustrated by the magnified $\alpha$-SMA details in Fig. 3c-e. In contrast, PCA-3DSIM is less sensitive to system errors and optical aberrations, resulting in higher-quality super-resolution results and improved optical sectioning performance. The fluorescence intensity distribution shown in Fig. 3f provides a more intuitive demonstration of the above conclusion, where PCA-3DSIM effectively suppresses the artifacts indicated by the green arrows, ensuring higher reconstruction fidelity. Supplementary file 5: Movie 4 provides the comparison between the 3D volumetric reconstruction results obtained using PCA-3DSIM and Open-3DSIM, respectively. To further evaluate the reconstruction quality of these two methods, the rolling Fourier ring correlation (rFRC) measurements^44^ of the reconstruction images from both PCA-3DSIM and Open-3DSIM were computed. These rFRC analyses enable the comparison of subtle improvements in reconstruction quality at the super-resolution scale and provide a robust metric for assessing the consistency of the reconstructions across different spatial frequencies. Figure 3g, h illustrate the PANEL (pixel-level analysis of error locations, which combines rFRC and truncated resolution-scaled error map^45^) maps of the super-resolution images obtained by the two methods for a certain layer, as well as the distributions of rFRC and PANEL values for all layers. In particular, the mean rFRC resolutions of specific lateral and axial slices in Fig. 3a were calculated. The statistical mean rFRC resolutions in the lateral direction for the super-resolution results of PCA-3DSIM and Open-3DSIM were 95.83 nm and 98.22 nm, respectively, while those in the axial direction were 296.95 nm and 298.04 nm, respectively. Although the improvement is moderate, these quantitative results suggest that PCA-3DSIM contributes to enhanced reconstruction quality. Moreover, when performing the adaptive tiled-block strategy, the speed and potential accuracy gains provided by the PCA-based approach render the reconstruction more tractable in practice.

### Experiments to demonstrate the high-quality reconstruction properties of PCA-3DSIM on data acquired by commercial SIM microscopes

**Fig. 4:**
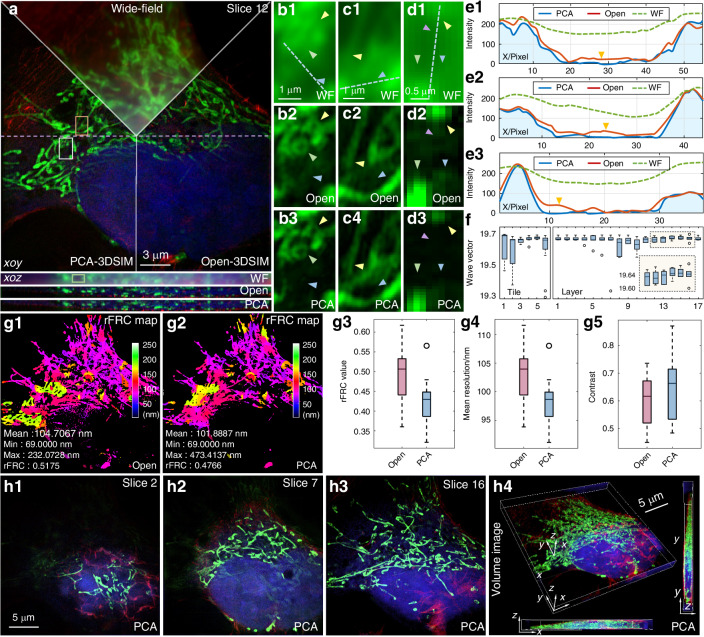
**Comparison of the multi-color super-resolution experimental results on a fixed COS-7 cell sample with DAPI-labeled nucleus, Alexa Fluor**^**TM**^ **568-labeled actin and MitoTracker**^**TM**^ **Green FM-labeled mitochondria**. **a** Comparison of the wide-field image and the super-resolution images obtained by different methods (Open-3DSIM^19^ and PCA-3DSIM), where the top are lateral slices of layer 12 and the bottom are axial slices along the purple dashed line. The raw SIM images were captured through a 100$\times$ objective (CFI Apochromat TIRF 100$\times$ Oil, NA 1.49, Nikon, Japan) of N-SIM (Nikon, Japan). For easy distinguishing, we show the mitochondria, actin and nucleus of the COS-7 cell in green, red and blue, respectively. The mitochondria are excited at 488 nm wavelength, the nucleus at 405 nm wavelength and the actin at 561 nm wavelength. **b**–**d** Magnified wide-field image and super-resolution images from the white-, yellow-, and green-boxed regions in (**a**) obtained by different methods. **e** Intensity profiles along the blue lines in (**b**)–(**d**). **f** The lateral wave vector distribution of the tiled images along the x-direction in a specific layer and that of the tiled images at a particular lateral position in different layers. g rFRC maps of super-resolved images obtained by different methods for a certain layer (g1 and g2), as well as the distribution of rFRC values (**g3**), mean resolutions (**g4**), and contrasts (**g5**) for 3D super-resolution stack images obtained by different methods. h Slices of super-resolution image stacks obtained by PCA-3DSIM in different layers as well as the whole 3D sight. The experiments were independently repeated 10 times with similar results. Colored arrows point to regions where reconstruction differences are distinct. Scale bars: 3 μm (**a**); 1 μm (**b**, **c**); 0.5 μm (**d**); 5 μm (**h**). Scale on z-axis: 17 layers, 0.125 μm per layer

Next the performance of PCA-3DSIM on data captured by commercial microscopes is tested. We utilized N-SIM to acquire layer-wise raw stack images of COS-7 cells in 3DSIM mode. Figure 4a illustrates the super-resolution images acquired by different methods (PCA-3DSIM and Open-3DSIM^19^). As seen in the magnified details of COS-7 mitochondria in Fig. 4b–d, PCA-3DSIM offers better spatial resolution than Open-3DSIM in the regions pointed by the yellow and green arrows, while suppressing artifacts interfering with reconstruction fidelity in the blue- and violet-arrowed regions, which can be more intuitively observed in the fluorescence intensity profile distribution shown in Fig. 4e. We provide in Fig. 4f the lateral wave vector distribution of the tiled images along the x-direction in a specific layer and that of the tiled images at a particular lateral position in different layers. These independent measurements of local reconstruction parameters contribute to the better properties of PCA-3DSIM. To more comprehensively assess the quality of super-resolution images obtained by different methods, we conducted rFRC measurements on the reconstructed 3D stack data, analyzing the rFRC values, mean resolutions, and image contrasts (Fig. 4g). These quantitative analyses further validate the better performance of PCA-3DSIM over Open-3DSIM. Figure 4g shows additional super-resolution images of different layers of COS-7 cells reconstructed by PCA-3DSIM, as well as the entire 3D view. Supplementary file 6: Movie 5 provides the comparison between the 3D volumetric reconstruction results obtained using PCA-3DSIM and Open-3DSIM, respectively. In addition, we applied PCA-3DSIM to specimens collected by different commercial SIM systems, such as US2O cells captured by N-SIM and OMX-SIM, respectively (Supplementary file 1: Figs. S6–S9). All these experimental results demonstrate that under the premise that commercial microscopes deliver more reliable mechanics and ensure fewer experimental imperfections, PCA-3DSIM still exhibits better reconstruction performance compared to the strategy that focuses parameter calculations on an individual layer, which also suggests that the spatial distortions mentioned above are prevalent and necessary to be targeted.

## Discussion

In summary, we have presented PCA-3DSIM, a novel 3DSIM reconstruction framework that extends PCA from 2D to 3D super-resolution microscopy. PCA-3DSIM employs PCA to extract reliable reconstruction parameters while integrating an adaptive tiled-block strategy to decompose the raw 3D stack into diverse volumetric tiled-block subsets. This combination effectively addresses spatial inconsistencies in experimental parameters caused by optical aberrations, system errors, and other factors. Simulation and experimental studies demonstrate that PCA-3DSIM delivers reliable reconstruction results and maintains image quality across different imaging platforms. In addition, the computational efficiency of PCA facilitates practical implementation, making PCA-3DSIM a flexible and accessible approach for high-quality, artifact-free 3D imaging of subcellular structures.

It should be emphasized that the current work mainly focuses on improving the parameter estimation accuracy of 3DSIM. The subsequent reconstruction of each subset is compatible with various deconvolution algorithms^19,21–25,40^, including those dealing with low SNRs. In this work, image reconstruction follows Open-3DSIM to ensure fair comparisons. In the image fusion stage, although the Sigmoid function-based weighting approach can suppress edge artifacts while ensuring structural fidelity, the overemphasis or attenuation of certain frequency information is unavoidable. Therefore, fusion algorithms that incorporate frequency-domain weighting can be explored to further enhance reconstruction quality. Regarding time consumption, although we apply PCA combined with a frequency-domain masking operator to compress the time cost of each parameter estimation while ensuring accuracy, a large number of repeated computations still lead to relatively high time consumption. Since the parameter solving for each subset is performed independently, it is suitable to further improve the processing efficiency by using CUDA, a framework provided by NVIDIA that enables parallel computation on Graphics Processing Units (GPUs) (detailed in Supplementary file 1: Note S5.3). Optimizing the algorithm and improving hardware efficiency will enable faster, potentially real-time, 3D super-resolution imaging of live cells, which remains a primary focus for future work.

## Materials and methods

### 3DSIM setup

Our custom 3DSIM system is implemented using an Olympus IX73 microscope with modified illumination and a nanoscale axial scanning stage (Supplementary file 1: Fig. S4). In the illumination light path, a series of dichroic mirrors (DM1: ZT561dcrb, Chroma, USA; and DM2: ZT488dcrb, Chroma, USA) are used to couple together lasers of different wavelengths (Laser 1: OBIS LX405, Coherent, USA; Laser 2: OBIS LX561, Coherent, USA; and Laser 3: Sapphire 488LP-200, Coherent, USA). The combined beam is first expanded and collimated using a spatial filter and an achromatic lens (L1: LSB08-A 150 mm, Thorlabs, USA). It then passes through a polarization beam splitter (PBS: PBS251, Thorlabs, USA), which is equipped with two half-wave plates (H1 and H2: GCL-0604, Daheng Optics, China). These wave plates help reduce energy loss caused by multiple reflections and transmissions. The beam then illuminates a ferroelectric liquid crystal spatial light modulator (SLM) that displays high-frequency fringe patterns. The generated diffracted light is focused by another achromatic lens (L2: LSB08-A 250 mm, Thorlabs, USA), and a carefully designed mask is used at the focusing plane to filter out the 0- and $\pm$1-order diffracted light. Usually, a spliced half-wave plate can be used to modulate the diffracted light to the polarization direction that optimizes the illuminated contrast. The diffracted light is relayed to the microscope by a lens group (L3: LSB08-A 200 mm, Thorlabs, USA; L4: LSB08-A 175 mm, Thorlabs, USA), and interferes at the sample to produce structured illumination. In the detection light path, the emitted sample fluorescence is imaged on the sCMOS camera (PCO Edge 5.5, PCO, Germany; with 60% quantum efficiency) target surface by the microscope objective (UPlanXApo 100$\times$/1.45 Oil, Olympus, Japan). At each layer along the axial direction, SLM sequentially displays the five-step phase-shifting fringe patterns in three different orientations (15 frames in total), and the sCMOS camera, the exposure of which can be triggered by SLM, acquires the resulting fluorescence images accordingly. By driving the nanoscale axial scanning stage (NanoScanz 200, Prior Scientific, UK), the acquisition of 3D raw stack images can be accomplished layer by layer.

### Calculation of modulation contrast-to-noise ratio (MCNR)

For a set of phase-shifting illumination images, the 1D Fourier transform is performed along the sequence of five phase shifts to obtain pixel-wise photon counts. The MCNR value is subsequently derived from the corresponding Fourier coefficients. These procedures can be realized by Eq. 2 and Eq. 3, respectively. Next, the MCNR values corresponding to the three illumination directions are averaged:

9 $$\overline{MCNR}=\frac{1}{3}\mathop{\sum }\limits_{\theta =1}^{3}MCN{R}_{\theta }$$

where the subscript $\theta$ represents different illumination directions. Among all pixels, the highest and the nearby second-highest values are identified to estimate the average MCNR for a set of illumination images:

10 $$av{g}_{MCNR}=\frac{1}{2}\{prctile[\overline{MCNR}(:,:),\,100]+prctile[\overline{MCNR}(:,:),\,100-a]\}$$

where ${\rm{prctile}}(X,p)$ represents the *p*-th percentile of dataset $X$, and $a$ is a constant ($5 < a < 10$). Finally, the average MCNR is normalized to evaluate the overall image quality of each focal slice:

11 $$MCN{R}_{norm}(z)=\frac{avg\_MCNR(z)}{\max (avg\_MCNR)}$$

where $MCN{R}_{norm}$ is the normalized MCNR of layer $z$, and $\max (\cdot )$ represents the operation to search for the maximum value. For the layers with normalized MCNR values higher than a certain threshold, they are considered to exhibit superior illumination image quality and can be directly applied to perform independent parameter estimation, while the parameters of the remaining layers are substituted as those estimated from the combined focal slices. For details on the determination of the MCNR threshold, see Supplementary file 1: Note S4.

### Nonlinear weight allocation method based on the Sigmoid function

Image fusion plays a critical role in determining the reconstruction quality of the final full FOV image, with improper processing potentially introducing biases. To suppress edge artifacts and preserve structural fidelity while maintaining computational efficiency, we adopt a Sigmoid-based nonlinear weighting strategy for full-FOV fusion.

For the fusion weight $\omega$ in Eq. 8, it can be obtained through the following formula:

12 $$\omega ({x}_{f})=\frac{1}{1+{e}^{-k({r}_{x}-{r}_{c})}}$$

where ${r}_{c}$ is the center point (inflection point) of the Sigmoid function, and $k$ denotes the parameter that controls the slope of the function (which is empirically set to 0.05). This approach offers several advantages. First, it effectively preserves detailed regions and eliminates edge artifacts by assigning higher weights to high-quality areas through the Sigmoid function. Moreover, it ensures smooth transitions at image boundaries and reduces noise accumulation by balancing the weights in overlapping regions. Finally, it is computationally efficient, as the Sigmoid weight map is generated once and can be reused across frames.

### Image processing

We provide two implementations for extending PCA-SIM to 3DSIM. If the illumination parameters are assumed to be uniformly distributed, the stack data (which can be regarded as a 3D tensor) in the regions where the +1- or -1-order spectral peaks are located are taken after the initial spectral separation and shifting of the raw stack data. The phasor of the Fourier inverse transform of this tensor is then obtained, and unfolded along the x-mode and y-mode, respectively. Since the unfolded matrix is essentially rank-1, PCA is utilized to acquire the first principal components.

The obtained singular value vectors can characterize the lateral component of the illumination parameter that filters out interfering noise. Subsequently, the axial component can be accessed through the angular relationship between the three amplitude wave vectors. With the exact illumination parameters, precise spectrum separation and reorganization can be performed, followed by super-resolution reconstruction. If uneven illumination parameters are to be handled, firstly, for the acquired raw 3D stack data with edge length $N$ and layer number ${N}_{z}$, it is divided into ${(\frac{2N}{m}-1)}^{2}$ tiled sub-stacks of size $2m\times 2m\times {N}_{z}$ (where $m$ is set to 64 to balance efficiency and accuracy), with half of the region of each sub-stack shared with its adjacent object. For any segmented tiled subset, the normalized MCNR, which characterizes the illumination modulation quality of the focal plane, is obtained by calculating the photon count for every pixel in the tiled image of each layer. The tiled images with MCNR exceeding a certain threshold (which is empirically taken to be 0.85) are considered capable of contributing reliable illumination parameters and are thus retained for subsequent processing. The remaining images in the sub-stack, however, are replaced by the composite image obtained through weighted averaging with relatively higher-quality images due to being in the defocused background. Next, the 3D reconstruction parameter distribution of the sub-stack is accessed layer by layer using a PCA-based parameter estimation approach. To further ensure the reliability of the calculated parameters, erroneous measurements are identified based on experimental prior knowledge. This prior assumes that wave vectors of different illumination directions are numerically close and differ by ~120° in orientation.

Once outliers are excluded, the parameters are recalculated using the full-FOV images. Using these experimental parameters, the super-resolution information of the sub-stack can be accurately demodulated and reconstructed (detailed in Supplementary file 1: Note S3.3). After performing deconvolution with the axial-extended OTF determined by the average lateral wave vector, the optimized super-resolution subset data can be obtained. The above steps are carried out for all tiled subsets, and finally, the fusion of each subset is accomplished by Eq. 6 to yield the full-FOV volumetric super-resolution information. Note that in most cases, the PCA-3DSIM version with the adaptive tiled-block strategy is recommended.

### Statistical analysis

Figures 2–4 show the representative data from 10 representative experiments. SSIM between the reconstruction results and the ground truth in Fig. 2, the PANEL and rFRC values of the reconstruction results in Fig. 3, and the distribution of the lateral illumination wave vectors in Fig. 4 are presented as box plots (center line, average; limits, 75% and 25%; whiskers, maximum and minimum) in graphs. The intensity profiles in Figs. 2–4 are in MATLAB.

## Supplementary information

Supplementary information

Movie S1. Illumination modulation in 3DSIM and the significance of illumination parameter estimation for super-resolution reconstruction

Movie S2. The principle of three-dimensional structured illumination microscopy based on principal component analysis (PCA-3DSIM) without the adaptive tiled-block strategy

Movie S3. The principle of three-dimensional structured illumination microscopy based on principal component analysis (PCA-3DSIM)

Movie S4. The comparison between the 3D volumetric reconstruction results of HeLa cells obtained using PCA-3DSIM and Open-3DSIM, respectively

Movie S5. The comparison between the 3D volumetric reconstruction results of COS-7 cells obtained using PCA-3DSIM and Open-3DSIM, respectively

## Data Availability

The customized MATLAB code of PCA-3DSIM is publicly available at https://figshare.com/s/93ef85f7b6f0a40f75f2.
