# Supplementary material for: Principal component analysis for three-dimensional structured illumination microscopy (PCA-3DSIM)
Source: Light Sci Appl. 2025 Sep 1;14:299. doi: 10.1038/s41377-025-01979-8 (PMC12402504; doi:10.1038/s41377-025-01979-8)
Supplement: Supplementary file 1 — Supplementary information [file 41377_2025_1979_MOESM1_ESM.pdf]

Supporting Information for
Principal component analysis for three-dimensional structured illumination microscopy (PCA-3DSIM)

Jiaming Qian[1,2,3,†], Weiyi Xia[1,2,3,†], Yuxia Huang[1,2,3], Jing Feng[1,2,3], Qian Chen[3,*], and Chao Zuo[1,2,3,*]

[1]Smart Computational Imaging (SCI) Laboratory, Nanjing University of Science and Technology, Nanjing, Jiangsu Province 210094, China
[2]Smart Computational Imaging Research Institute (SCIRI) of Nanjing University of Science and Technology, Nanjing, Jiangsu Province 210094, China
[3]Jiangsu Key Laboratory of Spectral Imaging & Intelligent Sense, Nanjing University of Science and Technology, Nanjing, Jiangsu Province 210094, China
[†]These authors contributed equally to this work
[*]chenqian@njust.edu.cn
[*]zuochao@njust.edu.cn

ABSTRACT

This document provides supplementary information for “Principal component analysis for three-dimensional structured illumination microscopy (PCA-3DSIM)”. We discuss in detail the algorithms for PCA-3DSIM, and present more comparison simulations and experiments to further demonstrate the performance of PCA-3DSIM. In addition, we provide an introduction to the open-source MATLAB code of PCA-3DSIM, as well as the corresponding user guide.

Contents

Supporting Information S1. The principle of three-dimensional structured illumination microscopy.
Supporting Information S2. Conventional 3DSIM reconstruction algorithm.
Supporting Information S3. 3D Parameter estimation based on principal component analysis and image reconstruction based on spectral optimization.
Supporting Information S4. Selections of the subset size and the MCNR threshold.
Supporting Information S5. Supplementary simulations and experiments.
Supporting Information S6. Supplementary code for PCA-3DSIM.
Supporting Information S7. Supplementary Videos.

Supporting Information S1. The principle of three-dimensional structured illumination microscopy

In fluorescence microscopy, the captured image $D(r)$ is the convolution of the emitted sample fluorescence $E(r)$ with the system point spread function (PSF):

$$D(r) = E(r) \otimes H(r) \tag{S1}$$

where $H(r)$ represents the system PSF. The Fourier transform of Eq. S1 can be expressed as:

$$\tilde{D}(k) = \tilde{E}(k)\tilde{H}(k) = \tilde{E}(k)O(k) \tag{S2}$$

where the superscript $\sim$ denotes the Fourier transform of the corresponding real-space quantity and $\tilde{H}(k)/O(k)$ is the system optical transfer function (OTF). The system OTF presents an annular support domain, and the "holes" in the annulus are the "missing cones" of information near the z-axis[1,2], as shown in Fig. S1a. The fluorescence image in Eq. S1 can be further expressed as:

$$E(r) = S(r)I(r) \tag{S3}$$

where $S(r)$ represents the object structure, *i.e.*, the density distribution of the fluorescent dye, and $I(r)$ is the excitation intensities. In two-dimensional structured illumination microscopy (2DSIM), the illumination pattern produced by two-beam interference can be expressed as:

$$I(r) = 1 + m \cdot \cos(2\pi pr) \tag{S4}$$

where m, p denote the modulation depth and wave vector of the illumination pattern, respectively. In this case, super-resolved signals of the sample that originally exceeded the diffraction limit in the lateral direction can be detected:

$$\tilde{D}(k) = \tilde{S}_0(k)O(k) + \frac{m}{2}\tilde{S}_{+1}(k-p)O(k) + \frac{m}{2}\tilde{S}_{-1}(k+p)O(k) \tag{S5}$$

where $\tilde{S}_0(k)O(k)$ represents the diffraction-limited wide-field information, and $\tilde{S}_{\pm 1}(k \mp p)O(k)$ represents super-resolved information beyond the diffraction limit. Note that Eq. S8 presents a sinusoidal distribution on the xoy plane, but remains consistent along the axial direction, so only lateral super-resolution can be achieved (Fig. S1b). In three-dimensional SIM (3DSIM), the illumination field generated by three-beam interference exhibits a sinusoidal distribution in both the lateral and axial directions, so it can be represented simplistically as a sum of a finite number of components, each of which can be divided into a product of an axial function and a lateral function:

$$I(r_{x,y}, z) = \sum_m I_m(z) J_m(r_{x,y}) \tag{S6}$$

where $r_{x,y}$ and z denote the spatial coordinates of the illumination field in the xoy and xoz planes, respectively, J_m is the transverse function, and I_m is the axial function. The modulated image can be

obtained by substituting Eq. S6 into Eq. S1:

$$D(r) = E(r) \otimes [S(r)I(r)] = \sum_m \int H(r-r')S(r')I_m(z')J_m(r'_{x,y})dr' \tag{S7}$$

where the coordinate r' is the sample reference system, the coordinate r is the dataset reference system, the axial coordinate z represents the physical displacement of the sample slide with respect to the objective lens, and the differential coordinate $(r-r')$ is the reference system of the objective lens. It can be seen that PSF depends on the differential coordinate $(r-r')$. If the illumination pattern is fixed on the focal plane of the objective lens during focusing, I_m will not depend on the citation coordinate z' but on the differential coordinate $(z-z')$ of the objective lens. Therefore, in the convolution integral, the axial portion of each illumination component is multiplied by PSF, not the sample $S(r)$:

$$D(r) = \sum_m \int H(r-r')I_m(z-z')S(r')J_m(r'_{x,y})dr' = \sum_m [H(r_{x,y},z)I_m(z)] \otimes [S(r_{x,y},z)J_m(r_{x,y})] \tag{S8}$$

Referring to Eq. S5, the term $S(r_{x,y},z)J_m(r_{x,y})$ in Eq. S8 transfers the high-frequency signals of the sample that originally exceeded the diffraction limit into the system detection support for lateral super-resolution, while $H(r_{x,y},z)I_m(z)$ extends the system OTF axially to achieve axial super-resolution (Fig. S1).

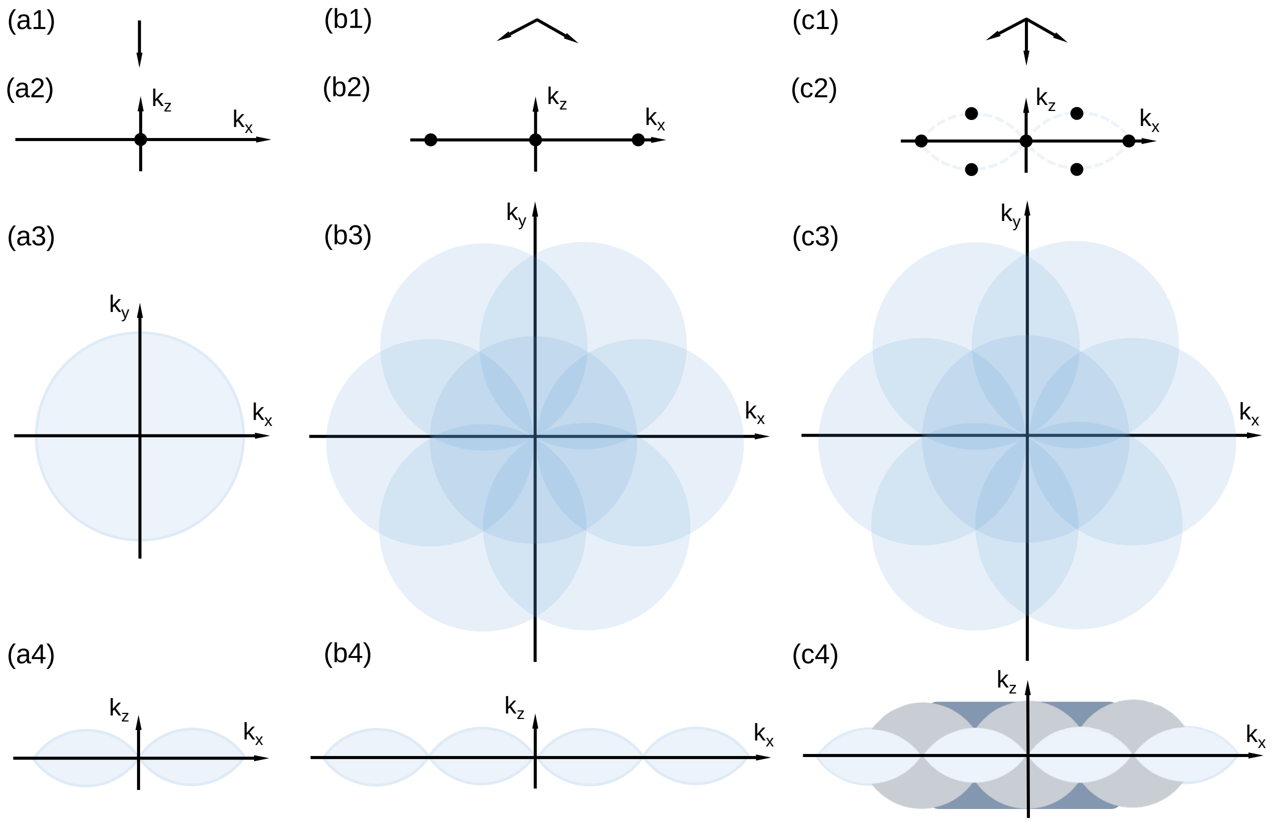

Figure S1. The principle of 3DSIM. (a) The amplitude wave vector, the spatial frequency component, and the projection of the 3D OTF in the k_x-k_y plane and the k_x-k_z plane, respectively, for the wide-field fluorescence mode. (b) The two amplitude wave vectors corresponding to the ±1-order diffraction beams, the spatial frequency components generated by two-beam interference, and the projection of the 3D OTF in the k_x-k_y plane and the k_x-k_z plane, respectively, for the wide-field fluorescence mode. (c) The three amplitude wave vectors corresponding to the 0- and ±1-order diffraction beams, the spatial frequency components generated by three-beam interference, and the projection of the 3D OTF in the k_x-k_y plane and the k_x-k_z plane, respectively, for the wide-field fluorescence mode.

Supporting Information S2. Conventional 3DSIM reconstruction algorithm

In 3DSIM, the illumination field generated by three-beam interference contains five pairs (seven in total) of frequency orders. In order to decouple the spectral components modulated by the illumination field, at least 5-step phase-shifting images need to be used in one illumination direction[3,4]. A certain illumination pattern sequence can be expressed as:

$$I_{\theta,\varphi}(r_{x,y},z) = I_0[1+2m^2+4m\cdot\cos 2\pi p_z z\cdot\cos(2\pi p_{x,y}\cdot r_{x,y}+\varphi)+2m^2\cos(4\pi p_{x,y}\cdot r_{x,y}+2\varphi)] \quad \text{(S9)}$$

where I_0 denotes the illumination intensity, m denotes the modulation depth of the focal plane, $p_{x,y}$ and p_z are the spatial frequencies of the illumination pattern in the xoy and xoz planes, $r_{x,y}$ is the vector in the xoy plane, θ and φ are the direction and phase of the illumination pattern, respectively. For simplicity, the illumination pattern is decomposed into a superposition of the zero, first and second harmonics, whose relative weights are $a_0 = 1+2m^2$, $a_{1z}(z) = 4m\cdot\cos 2\pi p_z z$ $(a_1 = 4m)$ and $a_2 = 2m^2$, respectively. After being illuminated by $I_{\theta,\varphi}(r_{x,y},z)$, the emitted fluorescence image $D_{\theta,\varphi}(r_{x,y},z)$ can be expressed as:

$$D_{\theta,\varphi}(r_{x,y},z) = \left[S(r_{x,y},z)\cdot I_{\theta,\varphi}(r_{x,y},z)\right]\otimes H(r_{x,y},z) \quad \text{(S10)}$$

The 3D frequency domain expression of Eq. S10 in the xoy plane can be expressed by $\tilde{D}_{\theta,\varphi}(k_{x,y},k_z)$:

$$\begin{aligned}\tilde{D}_{\theta,\varphi}(k_{x,y},k_z) &= \left[\tilde{S}(k_{x,y},k_z)\otimes\tilde{I}_{\theta,\varphi}(k_{x,y},k_z)\right]\cdot O(k_{x,y},k_z)\\ &= I_0\{a_0\cdot\tilde{S}(k_{x,y},k_z)+\tilde{a}_1(k_z)\cdot\frac{1}{2}[\tilde{S}(k_{x,y}-p_{x,y},k_z)e^{j\varphi}+\tilde{S}(k_{x,y}+p_{x,y},k_z)e^{-j\varphi}]\\ &+a_2\cdot\frac{1}{2}[\tilde{S}(k_{x,y}-2p_{x,y},k_z)e^{j2\varphi}+\tilde{S}(k_{x,y}+2p_{x,y},k_z)e^{-j2\varphi}]\}\cdot O(k_{x,y},k_z)\end{aligned} \quad \text{(S11)}$$

Ideally, the illumination pattern remains fixed with respect to the focal plane of the microscope and is sample independent. Thus, the axial function of the 3D illumination field can be transformed into a modulation of the system OTF to simplify subsequent operations. $a_{1z}(z)$ in the 1D frequency domain can be expressed as $\tilde{a}_1(k_z) = a_1\cdot\frac{1}{2}[\delta(k-p_z)+\delta(k+p_z)]$, which combined with the 3D frequency domain of the system OTF can be expressed as:

$$\tilde{a}_1(k_z)\cdot O(k_{x,y},k_z) = a_1\cdot\frac{1}{2}[O(k_{x,y},k_z-p_z)+O(k_{x,y},k_z+p_z)] \quad \text{(S12)}$$

By combining the five phase-shifting images, five spectral components $C_{0,\pm1,\pm2}(k)$ can be obtained:

$$\begin{bmatrix}C_0(k)\\C_{-1}(k)\\C_{+1}(k)\\C_{-2}(k)\\C_{+2}(k)\end{bmatrix} = \begin{bmatrix}a_0e^{j\varphi_0\cdot 0}\cdot\tilde{S}(k_{x,y},k_z)\cdot O(k_{x,y},k_z)\\ a_1e^{j\varphi_0\cdot(+1)}\cdot\tilde{S}(k_{x,y}-p_{x,y},k_z)\cdot\frac{1}{2}[O(k_{x,y},k_z-p_z)+O(k_{x,y},k_z+p_z)]\\ a_1e^{j\varphi_0\cdot(-1)}\cdot\tilde{S}(k_{x,y}+p_{x,y},k_z)\cdot\frac{1}{2}[O(k_{x,y},k_z-p_z)+O(k_{x,y},k_z+p_z)]\\ a_2e^{j\varphi_0\cdot(+2)}\cdot\tilde{S}(k_{x,y}-2p_{x,y},k_z)\cdot O(k_{x,y},k_z)\\ a_2e^{j\varphi_0\cdot(-2)}\cdot\tilde{S}(k_{x,y}+2p_{x,y},k_z)\cdot O(k_{x,y},k_z)\end{bmatrix} = \frac{W^{-1}}{I_0}\begin{bmatrix}D_{\theta,\varphi_1}(k)\\D_{\theta,\varphi_2}(k)\\D_{\theta,\varphi_3}(k)\\D_{\theta,\varphi_4}(k)\\D_{\theta,\varphi_5}(k)\end{bmatrix} \quad \text{(S13)}$$

where $W=\begin{bmatrix} 1 & \frac{1}{2}e^{j0} & \frac{1}{2}e^{-j0} & \frac{1}{2}e^{j2\cdot 0} & \frac{1}{2}e^{-j2\cdot 0} \\ 1 & \frac{1}{2}e^{j\frac{2}{5}\pi} & \frac{1}{2}e^{-j\frac{2}{5}\pi} & \frac{1}{2}e^{j2\cdot\frac{2}{5}\pi} & \frac{1}{2}e^{-j2\cdot\frac{2}{5}\pi} \\ 1 & \frac{1}{2}e^{j\frac{4}{5}\pi} & \frac{1}{2}e^{-j\frac{4}{5}\pi} & \frac{1}{2}e^{j2\cdot\frac{4}{5}\pi} & \frac{1}{2}e^{-j2\cdot\frac{4}{5}\pi} \\ 1 & \frac{1}{2}e^{j\frac{6}{5}\pi} & \frac{1}{2}e^{-j\frac{6}{5}\pi} & \frac{1}{2}e^{j2\cdot\frac{6}{5}\pi} & \frac{1}{2}e^{-j2\cdot\frac{6}{5}\pi} \\ 1 & \frac{1}{2}e^{j\frac{8}{5}\pi} & \frac{1}{2}e^{-j\frac{8}{5}\pi} & \frac{1}{2}e^{j2\cdot\frac{8}{5}\pi} & \frac{1}{2}e^{-j2\cdot\frac{8}{5}\pi} \end{bmatrix}$.

In order to recover the high-frequency information, the five spectral components need to be shifted to their correct locations, which requires further accurate estimation of the wave vector $p_{x,y}$ and the initial phase φ_0 (these illumination parameters are usually acquired in a 2D manner from a set of lateral images with optimal illumination modulation quality or synthesized images, treated as constants[5,6]):

$$C_{ns}(k)=F\left\{F^{-1}\left[C_n(k)\right]\cdot e^{-jn2\pi p_{x,y}}\right\} \tag{S14}$$

where n denotes the frequency order (n=0,±1,±2), and $F\{\cdot\}$ ($F^{-1}\{\cdot\}$) represent the 3D (inverse) Fourier transform operation. The shifted spectrum components can be expressed as:

$$\begin{bmatrix} C_{0s}(k) \\ C_{-1s}(k) \\ C_{+1s}(k) \\ C_{-2s}(k) \\ C_{+2s}(k) \end{bmatrix} = \begin{bmatrix} e^{j\varphi_0\cdot 0}\cdot\tilde{S}(k_{x,y},k_z)\cdot O_{0s}(k) \\ m_1 e^{j\varphi_0\cdot(+1)}\cdot\tilde{S}(k_{x,y},k_z)\cdot O_{-1s}(k) \\ m_1 e^{j\varphi_0\cdot(-1)}\cdot\tilde{S}(k_{x,y},k_z)\cdot O_{+1s}(k) \\ m_2 e^{j\varphi_0\cdot(+2)}\cdot\tilde{S}(k_{x,y},k_z)\cdot O_{-2s}(k) \\ m_2 e^{j\varphi_0\cdot(-2)}\cdot\tilde{S}(k_{x,y},k_z)\cdot O_{+2s}(k) \end{bmatrix} \tag{S15}$$

where $O_{ns}(k)$ can be expressed as $\begin{bmatrix} O_{0s}(k) \\ O_{-1s}(k) \\ O_{+1s}(k) \\ O_{-2s}(k) \\ O_{+2s}(k) \end{bmatrix} = \begin{bmatrix} O(k_{x,y},k_z) \\ \frac{1}{2}\left[O(k_{x,y}+p_{x,y},k_z-p_z)+O(k_{x,y}+p_{x,y},k_z+p_z)\right] \\ \frac{1}{2}\left[O(k_{x,y}-p_{x,y},k_z-p_z)+O(k_{x,y}-p_{x,y},k_z+p_z)\right] \\ O(k_{x,y}+2p_{x,y},k_z) \\ O(k_{x,y}-2p_{x,y},k_z) \end{bmatrix}$.

Finally, the super-resolution image can be obtained by recombining the spectrum components $C_{ns}(k)$ through Wiener deconvolution.

$$\widehat{\tilde{S}}(k)=\frac{\sum_{d,n}O_{ns}^{*}(k)\,C_{ns}(k)}{\sum_{d,n}|O_{ns}(k)|^2+w^2}\cdot apo(k) \quad (d=1,2,3;n=0,\pm1,\pm2) \tag{S16}$$

where $\widehat{\tilde{S}}(k)$ represents the estimate of the true sample $\tilde{S}(k)$, w^2 denotes the Wiener parameter, and $apo(k)$ is the apodization function.

Supporting Information S3. 3D Parameter estimation based on principal component analysis and image reconstruction based on spectral optimization

In order to obtain 3D illumination parameter distributions, the structured illumination parameter estimation approach based on principal component analysis (PCA)[7] is extended from 2DSIM to 3DSIM, which ensures the measurement accuracy while alleviating the time consumption due to the increase in data volume.

S3.1 Parameter estimation based on high-order principal component analysis

Taking the -2-order spectral component $C_{-2}(k)$ as an example, it can be represented after performing the integer-pixel shift as follows:

$$C_{-2}(k)_{shift} = a_2 e^{j\varphi_0\cdot(+2)} \cdot \tilde{S}(k_{x,y} - p_{sub}, k_z) \cdot O(k_{x,y} + p_{int}, k_z) \tag{S17}$$

where p_{int} and p_{sub} denote the integer-pixel and sub-pixel portions of the wave vector p ($p = 2p_{x,y} = p_{int} + p_{sub}$). The inverse Fourier transform of $C_{-2}(k)_{shift}$ can be expressed as follows if the effects of modulation depth and OTF are neglected:

$$\mathscr{F}^{-1}\left[e^{j\varphi_0\cdot(+2)} \cdot \tilde{S}(k_{x,y} - p_{sub}, k_z)\right] = S(r_{x,y}, z)\, e^{j\left(p_{sub}\cdot r_{x,y} + 2\varphi_0\right)} \tag{S18}$$

where $\mathscr{F}^{-1}[\cdot]$ denotes the inverse Fourier transform, and $r_{x,y}$ and z denote the spatial coordinates in the lateral and axial directions respectively. For the 3D phasor term $e^{j[p_{sub}(r_{x,y},z)+2\varphi_0]}$ of the stack data, it is not difficult to find that the slope and constant term of its phase expression are just the sub-pixel wave vector p_{sub} and the initial phase $2\varphi_0$. However, due to factors such as noise, optical aberration, and other interfering factors, the phasor obtained experimentally ($e^{i\cdot angle\{\mathscr{F}^{-1}[C_{-2}(k)_{shift}]\}}$, where $angle(\cdot)$ denotes the operation of extracting the phase) can be seriously disturbed, making it difficult to obtain the accurate illumination parameters. For simplicity, $e^{j[p_{sub}(r_{x,y},z)+2\varphi_0]}$ can be characterized as a 3-order tensor $\mathscr{I} \in R^{N_x\times N_y\times N_z}$ (where N_n represents the data length on the n-axis). From the perspective of data features, any lateral and axial slice of tensor $\mathscr{I}$ can be decomposed into the product of two orthogonal vectors according to the exponent addition property, implying that the Tucker-rank of $\mathscr{I}$ in any mode is one, *i.e.*, matrices unfolded from $\mathscr{I}$ along different modes are essentially rank-one[8]. Based on the above analysis, the ideal phasor tensor can be attained from the first principal component extracted by high-order singular value decomposition (HOSVD)[9,10] of $e^{i\cdot angle\{\mathscr{F}^{-1}[C_{-2}(k)_{shift}]\}}$:

$$\mathscr{I}(x,y,z) \approx \mathscr{T} \times_x U^{(x)} \times_y U^{(y)} \times_z U^{(z)} \tag{S19}$$

where $\times_n$ represents the n-mode product operation, $\mathscr{T}$ is the core tensor, which retains in each mode only the dimensions consistent with the Tucker-rank of $\mathscr{I}$, and $U^{(n)}$ denotes the left singular matrix of the n-mode unfolding matrix. Considering the x-mode unfolding matrix $\mathscr{I}_{(x)} \in R^{N_x\times(N_y\cdot N_z)}$, it can be further expressed as:

$$\mathscr{I}_{(x)} = s_x s_{y,z}^H \tag{S20}$$

$$s_{y,z} = [s^T_{y(z=1)} \left| \cdots s^T_{y(z=N_z-1)} \right| s^T_{y(z=N_z)}]^T \tag{S21}$$

where $s_x = e^{j(p_{x,sub} \cdot r_x + 2\varphi_{x,0})}$, $p_{x,sub}$ and $\varphi_{x,0}$ are the x-direction components of the wave vector and the initial phase, $s_{y,z}$ is a vector of similar form unfolded along the z-direction, and $\{\cdot\}^T$ and $\{\cdot\}^H$ represent the transpose and complex conjugate transpose operations of the original object respectively. Since the unfolding matrix is rank-one, s_x can be directly characterized by the singular vector corresponding to the first principal component in $U^{(x)}$, meaning that $p_{x,sub}$ and $\varphi_{x,0}$ are conveniently available. Therefore, by simply performing linear regression on the principal singular vectors of $U^{(x)}$ and $U^{(y)}$, the precise illumination parameters (p_{sub} and φ_0), with noise and other high-dimensional data components filtered out, can be acquired.

S3.2 3D parameter estimation based on principal component analysis

When the adaptive tiled-block strategy is applied, the frequency-shifted -2-order spectrum of an any tiled image can be expressed as follows after ignoring the influence of the system OTF and the modulation depth:

$$\mathscr{F}^{-1}\left[e^{j\varphi_0 \cdot (+2)} \cdot \tilde{S}\left(k_{x,y} - p_{sub}, k_z\right)\right] = S\left(r_{x,y}, z\right) e^{j\left(p_{sub} \cdot r_{x,y} + 2\varphi_0\right)} \tag{S22}$$

where $e^{j\left(p_{sub} \cdot r_{x,y} + 2\varphi_0\right)}$ is the 2D phasor matrix of the illumination pattern, and the slope and constant term of its plane are the sub-pixel wave vector p_{sub} and the initial phase $2\varphi_0$. Further analysis of the ideal illumination pattern phasor matrix $e^{j\left(p_{sub} \cdot r_{x,y} + 2\varphi_0\right)}$ reveals that it can be decomposed into the product of two vectors:

$$e^{j\left(p_{sub} \cdot r_{x,y} + 2\varphi_0\right)} = s_x s_y{}^H \tag{S23}$$

where $s_x = \exp[j(p_{x,sub} \cdot r_x + 2\varphi_{x,0})]$ and $s_y = \exp[j(p_{y,sub} \cdot r_y + 2\varphi_{y,0})]$. Such decomposition reveals that the ideal pattern phasor matrix is essentially a rank-one matrix, which has only one principal component describing the single best subspace in the least squares sense, while the experimental imperfections and other disturbances result in high dimensionality of the pattern phasor matrix. Then the task of searching for p_{sub} is translated into solving for the rank one approximation subspace of $\exp\{j \cdot angle[\mathscr{F}^{-1}(C_{-2}(k)_{shift})]\}$. Therefore, PCA is utilized to remove the interfering components from the experimentally captured illumination phasor matrix and extract the "first principal component" dominated by the illumination parameters. Specifically, the eigenvalues of the actual illumination phasor matrix are obtained by singular value decomposition (SVD):

$$\exp\{j \cdot angle[\mathscr{F}^{-1}(C_{-2}(k)_{shift})]\} = U \Lambda V^T \tag{S24}$$

where Λ is a semi-positive definite diagonal matrix whose elements on the diagonal are the eigenvalues of the illuminated phasor matrix, U and V are the unitary matrices consisting of the left and right singular vectors, respectively, and the superscript T denotes the transpose operation on the matrices. Then the left and right singular vectors corresponding to the first principal component obtained by SVD are phase

expanded and least squares fitted along the x-axis and y-axis, respectively, in one dimension. The slopes ($p_{x,sub}$ and $p_{y,sub}$) and the constant terms ($2\varphi_{x,0}$ and $2\varphi_{y,0}$) of the fitted linear expressions are subpixel portions of the illumination wave vectors and initial phases to be solved.

However, at very low signal-to-noise ratios (SNRs), irrelevant interference can still affect the accuracy of parameter estimation. In addition, performing PCA on a large amount of data is time-consuming. In fact, the Fourier spectrum of an ideal pattern phasor matrix $e^{j\left(p_{sub}\cdot r_{x,y}+2\varphi_0\right)}$ is a downsampled 2D Dirichlet function[11] with most of its effective energy is concentrated in limited support around the integer part of the wave vector. Based on this property, a frequency-domain dual-window mask operator containing an inner signal window and an external padding window is further introduced to accelerate PCA-related operations and improve noise robustness before applying PCA. Specifically, the high-energy spectral peak region is retained through the signal window, while noisy data outside this window are set to zero:

$$C_{-2}(k)_{shjft} = \begin{cases} a_2 e^{j\varphi_0\cdot(+2)}\cdot\tilde{S}_{-2}\left(k_{x,y}-p_{sub},k_z\right)\cdot O\left(k_{x,y}+p_{int},k_z\right) & k_{x/y,\min}\le k_{x/y}\le k_{x/y,\max} \\ 0 & \begin{matrix} k_{x/y,\min}-R\le k_{x/y}<k_{x/y,\min} \\ k_{x/y,\max}\le k_{x/y}<k_{x/y,\max}+R \end{matrix} \\ NaN & \begin{matrix} k_{x/y}<k_{x/y,\min}-R \\ k_{x/y}>k_{x/y,\max}+R \end{matrix} \end{cases} \tag{S25}$$

where $k_{x,\min}$, $k_{y,\min}$ denote the left boundary or the lower boundary of the signal window in the frequency-domain masking operator in the horizontal or vertical direction, $k_{x,\max}$, $k_{y,\max}$ denote the right boundary or the upper boundary of the signal window in the horizontal or vertical direction, R represents the width of the padding window surrounding the signal window, and k_x, k_y represent the frequency coordinates along the horizontal and vertical directions. Since the limited mask contains almost the main energy of the downsampled 2D Dirichlet function, and the noise distributed over the full field range is significantly suppressed, the accuracy and robustness of the parameter estimation will be significantly improved. In addition, the masking operator significantly reduces the data amount involved in the PCA calculation, thereby dramatically improving the computational efficiency of the whole algorithm.

S3.3 Image reconstruction based on spectral optimization

To cope with spectral anomalies and various artifacts, a series of frequency-domain filters in Open-3DSIM[5] are employed to optimize the reconstructed spectrum.

For the honeycomb artifacts caused by the out-of-focus background and other factors, usually notch filters are applied to suppress high-frequency peaks[12]:

$$C_{SR\_1}(k) = \sum_{n=-2}^{2} C_{ns}(k)\cdot noth(x,y,z,n)\cdot OTF^{att}(x,y,z,n) \tag{S26}$$

$$noth(x,y,z,0) = 1-d\cdot\exp[(\frac{x^2+y^2}{\left|p_{xy}\right|^2}+\frac{z^2}{\left|p_z\right|^2})/2/w^2] \tag{S27}$$

where $noth(x,y,z,0)$ is the notch filter designed based on the estimated illumination wave vectors, $noth(x,y,z,n)$ is the frequency-shifted version based on $noth(x,y,z,0)$, $OTF(x,y,z,n)$ denotes the shifted

3D OTF corresponding to the n-order spectrum information, att is the frequency attenuation, and d and w represent the notch width and depth, respectively.

Since the above notch filters will also damage the high-frequency data of the sample while removing the honeycomb artifacts, a spatial sum of $OTF \cdot notch$ is designed to further optimize the structured spectrum:

$$OTF_{notch} = \sum_{n=-2}^{2} m(n) \cdot OTF(x,y,z,n) \cdot noth(x,y,z,n) \tag{S28}$$

where $m(n)$ is the weight coefficient of different Fourier orders. Considering that the ideal spectrum of 3DSIM is smooth in the volume space, $Filter1(k) = \frac{Apo}{OTF_{notch}+{w_1}^2}$ is adopted to correct for anomalous frequencies while suppressing patchy features and high-frequency noise:

$$C_{SR\_2}(k) = C_{SR\_1}(k) \cdot \frac{Apo}{OTF_{notch} + {w_1}^2} \tag{S29}$$

where Apo is the apodization function in the 3D frequency domain and w_1 is the design parameter of $Filter1(k)$. Finally, an additional filter $Filter2(k) = \frac{Apo}{OTF_{notch}+{w_2}^2}$ is designed to make the reconstructed spectrum close to its ideal form and preserve the weak information of the reconstructed image:

$$C_{SR\_3}(k) = C_{SR\_3}(k) \cdot \frac{Apo}{OTF_{notch} + {w_2}^2} \tag{S30}$$

where w_2 is relatively smaller than w_1 with the purpose of increasing the proportion of high-frequency component. The cooperation of $Filter1(k)$ and $Filter2(k)$ can fill the spectral holes caused by the above-mentioned notch operations and improve the smoothness of the reconstructed spectrum. The final optimized super-resolution images can be obtained through the inverse Fourier transform of $C_{SR\_3}(k)$. For the selection of some parameters such as d, w, w_1, w_2, *etc.* in the above equations, please refer to this literature[5].

Supporting Information S4. Selections of the subset size and the MCNR threshold

S4.1 Selection of the subset size

The choice of subset size is crucial for the accuracy and efficiency of illumination parameter measurements. On the one hand, smaller sizes ensure fewer parameter variations over the range in a single tiled image. On the other hand, excessively small dimensions can lead to a significant computational burden. To determine a reasonable subset size that balances efficiency and accuracy, we use illumination parameters obtained from subsets of different sizes to reconstruct super-resolved images. The tested data are fluorescence images (with size of 512×512×17 pixels) of COS-7 cells acquired by a commercial SIM microscope (N-SIM, Nikon, Japan) in 3DSIM mode.

As illustrated in Fig. S2, Fig. S2a shows the super-resolution results obtained by Open-3DSIM[5] (one of the state-of-the-art 3DSIM reconstruction methods, which estimates illumination parameters from layer data superimposed by patterns with high modulation quality), and Figs. S2b-S2e are results of PCA-3DSIM using the same data but with progressively smaller tile sizes. It can be seen that when the tile edge length is reduced to 128 pixels, there is a significant improvement in reconstruction quality (Figs. S2c and S2d). However, very small tiles result in pixelation artefacts in the final reconstructed images, as seen in both real-space images (regions marked in yellow in Fig. S2e3) and Fourier-space images (regions marked in yellow in Figs. S2d4 and S2e4). This is due to the fact that with smaller tile sizes, the sample count is too low, leading to PCA overfitting, where the extracted principal components may not accurately represent the true structure of the data. Additionally, in the parameter estimation of extremely small tiles, the selected tiled subregions may contain invalid background information, which can cause abnormal parameter estimation.

Regarding the overlap rate between neighboring tiles, tile reconstruction is insensitive to the amount of overlap, which is chosen to be half of the tile width in order to suppress boundaries and artifacts caused by image fusion as much as possible. Therefore, taking into account both the reconstruction efficiency and accuracy, in this study, we use 128-pixel tiles with 64-pixel overlays for PCA-3DSIM.

In order to more intuitively visualize the impact of tile size selection on the reconstruction performance, we list the image reconstruction qualities as well as time consumptions for different tile sizes in Table S1. We utilize the labels '$\surd$', '$-$' and '$\times$' to represent good, fair, and compromised relative performance, with respect to different properties. In addition, the performance level can also be reflected by the shade of the background color, with darker hues representing better performance. Users can refer to Table S1 to select appropriate parameters for different application requirements. It is worth noting that the time consumption listed in Table S1 is based on unoptimized computations. Further improvements in algorithm design, along with the integration of parallel computing techniques, can significantly reduce the computation time (detailed in Section S5.3).

S4.2 Selection of the MCNR threshold

Regarding the selection of the threshold for the modulation contrast-to-noise ratio (MCNR), it is designed to assess whether the quality of the tiled image set at a certain layer is sufficient to support high-precision parameter estimation. Theoretically, a higher MCNR threshold imposes stricter quality control. As the MCNR value decreases, the parameters obtained from layer data are gradually replaced by those derived from block-merged data. However, when the threshold is set too high, there may be cases where the MCNR value is relatively large but still below the threshold. In such cases, although the quality of layer-based parameters is not optimal, they may still be more suitable for reconstructing that particular layer compared to block-based parameters. Therefore, a reasonable choice of the MCNR threshold is crucial to ensuring the reconstruction quality.

As illustrated in Fig. S3, we evaluated the reconstruction performance of PCA-3DSIM under different MCNR thresholds ranging from 0.7 to 0.95, with a step size of 0.05. It can be seen that, as the MCNR threshold increases (from 0.7 to 0.85), the defocus artifacts in the regions indicated by blue and green arrows gradually diminish. However, further increasing the threshold (from 0.85 to 0.95) leads to a resurgence of artifacts, as shown in the blue-marked regions of Figs. S3a-S3c. Figure S3g provides a clearer characterization of the relationship between the MCNR threshold and reconstruction performance. It reveals that the overall reconstruction quality reaches its optimum when the MCNR threshold is set around 0.85.

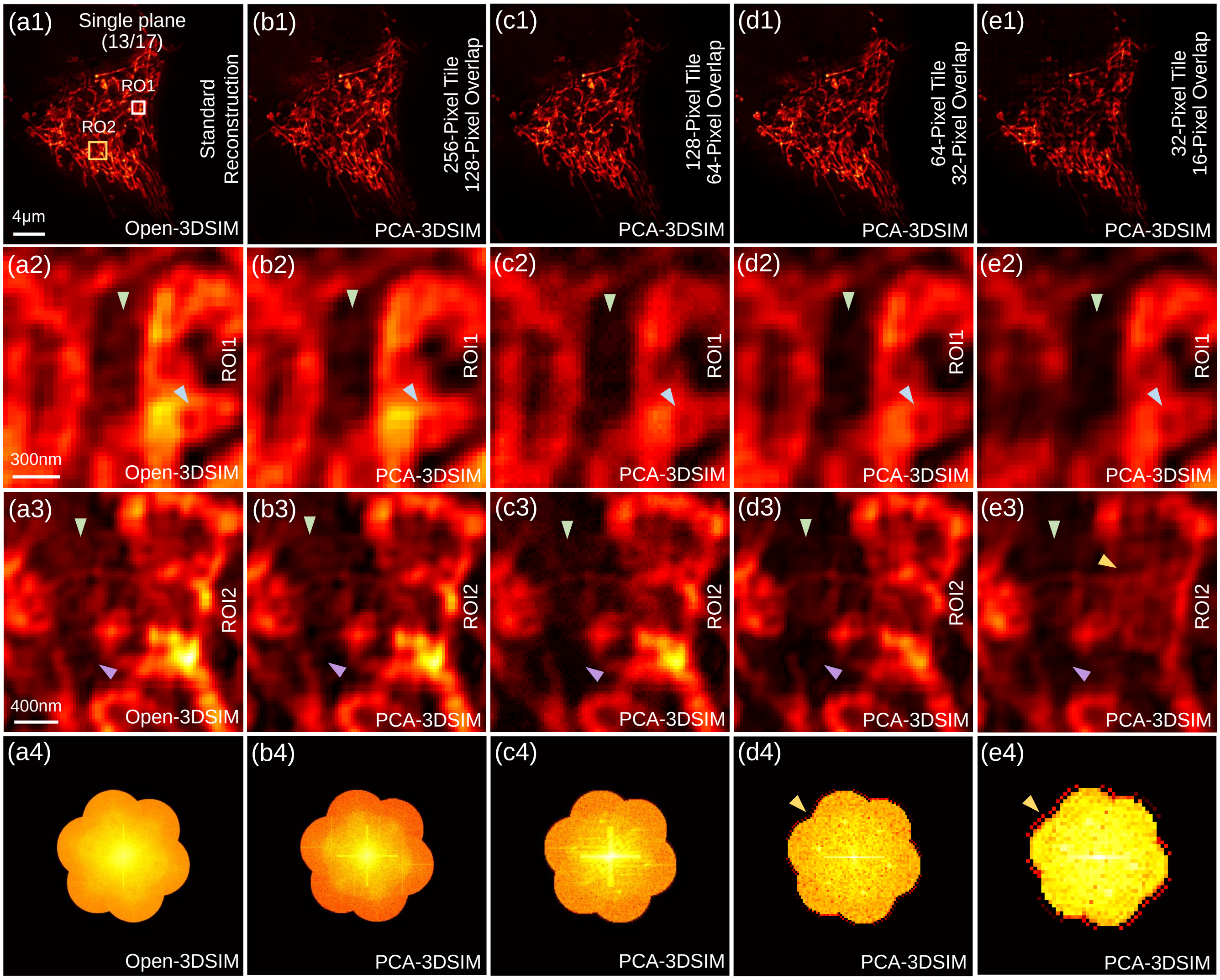

Figure S2. Reconstruction results using PCA-3DSIM with different tile sizes. (a1)-(e1) Full field-of-view (FOV) super-resolution images obtain by Open-3DSIM[5] and PCA-3DSIM with different tile sizes for a specific layer. (a2)-(e2) Magnified super-resolution images from the white-boxed regions in (a1)-(e1) obtained by different methods (Open-3DSIM[5] and PCA-3DSIM). (a3)-(e3) Magnified super-resolution images from the yellow-boxed regions in (a1)-(e1) obtained by different methods. (a4)-(e4) Spectrum images of the full-FOV or tile reconstruction images obtained by different methods. The experiments were independently repeated 10 times with similar results. Colored arrows point to regions where reconstruction differences are distinct. Scale bars: 4 μm (a1); 300 nm (a2); 400 nm (a3).

Table S1. Effect of different tile sizes on reconstruction quality and processing speed. ‘$\surd$’, ‘$-$’ and ‘$\times$’ indicate good, fair and compromised performance with respect to different properties. Regarding color, the darker the background color, the better the corresponding performance.

Tile size	Overlap size	Reconstruction quality	Time consumption	
512×512 pixels (Full FOV)	/	×	230.143734 s	√
256×256 pixels	128×128 pixels	—	633.169595 s	—
128×128 pixels	64×64 pixels	√	810.390414 s	—
64×64 pixels	32×32 pixels	√ (Slight pixelation artifacts)	1425.278464 s	×
32×32 pixels	16×16 pixels	× (Severe pixelation artifacts)	1965.377492 s	×

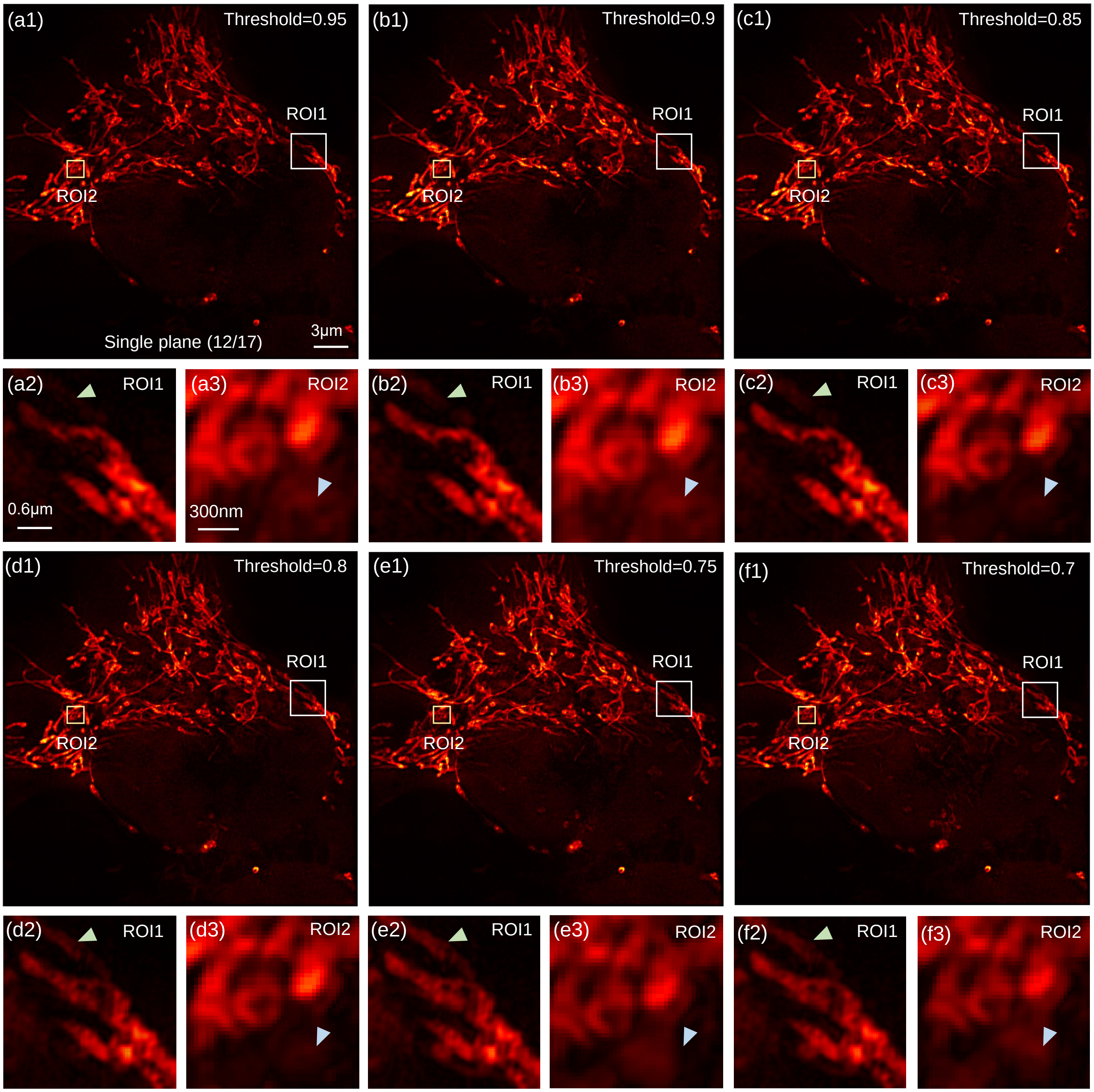

(g) MCNR threshold	0.95	0.90	0.85	0.80	0.75	0.70
Reconstruction quality	—	—	√	—	×	×

Figure S3. Reconstruction results using PCA-3DSIM with different MCNR thresholds. (a)-(f) Full FOV super-resolution images obtained by PCA-3DSIM with different MCNR thresholds for a specific layer. (g) Effect of different MCNR thresholds on reconstruction quality. '$\surd$', '$-$' and '$\times$' indicate good, fair and compromised performance. Regarding color, the darker the background color, the better the corresponding performance. The experiments were independently repeated 10 times with similar results. Colored arrows point to regions where reconstruction differences are distinct. Scale bars: 3 μm (a1); 600 nm (a2); 300 nm (a3).

Supporting Information S5. Supplementary simulations and experiments

S5.1 Supplementary simulations

We provide more simulation results to verify the effectiveness of PCA-3DSIM. As shown in Fig. S4a, we reconstruct a region with high modulation contrast to noise ratio (MCNR) values using Open-3DSIM, hPCA-3DSIM, and alPCA-3DSIM, respectively, where hPCA-3DSIM refers to the version of PCA-3DSIM without the adaptive tiled-block segmentation. As can be seen, both Open-3DSIM and hPCA-3DSIM obtain resolution-enhanced results of similar quality, but suffer from some artifacts, as shown in the area indicated by the green arrows. Based on hPCA-3DSIM, further implementation of the layer-wise estimation strategy (*i.e.*, alPCA-3DSIM) enables acquiring axially distributed illumination parameters, which prompts higher-quality super-resolution reconstructions. In regions with low MCNR values, parameter estimation based solely on the corresponding layer may be less reliable than that derived from the optimal layer or the fused layer. In this case, a block-wise merging strategy is employed to enhance the robustness and accuracy of parameter estimation. As shown in Fig. S4b, we present the super-resolution results for regions where the MCNR value is less than the threshold used to judge the modulation quality. If the data from a single layer is still employed (*i.e.*, lPCA-3DSIM, which relies directly on the images from a single layer to obtain illumination parameters), artifacts would occur in the reconstruction results. In contrast, after adaptively replacing the data originally used for parameter estimation with synthetic versions of the different layers, the reconstruction quality is significantly improved. The above strategies only account for axial variations in the reconstruction parameters. By further incorporating tiled reconstruction, spatial variations across the entire volume can be jointly addressed, thereby improving the overall reconstruction quality. As seen in Fig. S4c, compared to alPCA-3DSIM, PCA-3DSIM with the adaptive tiled-block strategy reconstructs higher quality super-resolution images with further tile segmentation. In fact, the adaptive tiled-block strategy can be regarded as a generalized approach capable of improving the reconstruction quality of the original reconstruction method. To verify this, we applied the adaptive tiled-block strategy to Open-3DSIM (*i.e.*, tOpen-3DSIM) and obtained results that outperform alPCA (Fig. S4c). However, Open-3DSIM mainly relies on the cross-correlation (COR)-based method for parameter estimation, which is slightly inferior to PCA[7] in terms of accuracy and noise robustness. As a result, its reconstructions still exhibit additional image artifacts compared to those of PCA-SIM. In addition, the COR-based approach is nearly 20 times more time-consuming than PCA[7], further increasing the overall computational cost (this is exacerbated by the segmentation operation). Regarding the fusion of subsets, as shown in Fig. S4d, after using the weighted average fusion algorithm, the boundaries between neighboring sub-images are eliminated, resulting in high-quality super-resolution data with full FOV. In addition, we calculated the structural similarity coefficient index (SSIM) between the tile images before and after image fusion and the ground truth images. As illustrated in Fig. S4e, the SSIM values of the fused images are significantly better than those of the unfused versions, effectively maintaining the structural fidelity.

S5.2 Supplementary experiments

Next, we supplement the comparative super-resolution results on data obtained from different 3DSIM systems to further validate the effectiveness of PCA-3DSIM. Figure S6 illustrates the super-resolution results of actin images of U2OS cells obtained by N-SIM (Nikon, Japan) using different methods (Open-3DSIM[5], hPCA-SIM, tOpen-3DSIM and PCA-3DSIM). As seen, all these approaches attain high-quality super-resolution images with improved resolution (Fig. S6a). Further observation of the magnified regions of interest indicates that, compared to Open-3DSIM and hPCA-3DSIM, both tOpen-3DSIM and

PCA-3DSIM offer enhancements in both super-resolution performance (regions pointed by green arrows in Fig. S6c) and optical sectioning capability (regions pointed by blue and yellow arrows in Figs. S6b and S6d) after applying the adaptive tiled-block strategy to takes into account the 3D illumination parameter distribution. In addition, the reconstruction quality obtained by PCA-3DSIM is slightly better than that of tOpen-3DSIM as the PCA method can achieve higher parameter estimation accuracy and noise immunity than the cross-correlation method[7] (regions pointed by purple arrows in Figs. S6b and S6c). Figures S7-S9 provide more comparison experiments, including results for U2OS actin acquired by OMX-SIM at low SNRs[5] as well as those for live sporulating Bacillus subtilis acquired by custom 3DSIM microscopy[6]. All these experimental results indicate that by combining PCA with the adaptive tiled-block strategy, reliable 3D illumination parameters can be effectively obtained, thereby achieving higher-quality super-resolution reconstruction performance compared to conventional methods.

S5.3 Time consumption of PCA-3DSIM and algorithm acceleration scheme

Taking the original images ($512\times512\times10$) in Fig. S7 as an example, the execution time of each key step, obtained by running our open-source code in MATLAB R2018a on a Dell computer (Intel(R) Core(TM) i7-9700 CPU, NVIDIA GeForce GTX 1660 Ti), is listed in Table S2. It can be seen that performing a round of MCNR value computation and PCA-based parameter estimation is not time-consuming, especially the parameter estimation, both of which run in the millisecond level. However, the repeated computations caused by the adaptive segmentation strategy seriously impact the overall runtime. For MCNR calculation, the large number of pixel-wise operations make it well-suited for CUDA-based parallel acceleration on Graphics Processing Units (GPUs), which can significantly improve computational efficiency. For PCA-based parameter estimation, since each tile needs to be parameterized independently, multi-thread parallel execution can be employed to avoid the additional time cost caused by redundant computations. For image reconstruction, the frequency-domain filters are applied to the reconstructed spectrum in a pixel-wise manner, which can be easily optimized through GPU parallel processing. Meanwhile, the reconstructions of multiple blocks can be performed in parallel across multiple threads. For image fusion, the Sigmoid-based weighting method inherently supports GPU-parallel computation between adjacent tiled images. Along the fusion path, the row-wise operations can be decomposed into computations proceeding simultaneously from the top and bottom rows toward the center, thus supporting dual-thread parallel path optimization. For the basic operations of Fourier transform and inverse Fourier transform, CUDA provides fundamental functions that enable fast computation on GPUs. As for the system OTF used for deconvolution, it can be obtained through calibration or simulation, allowing it to be pre-generated without occupying the overall computation time.

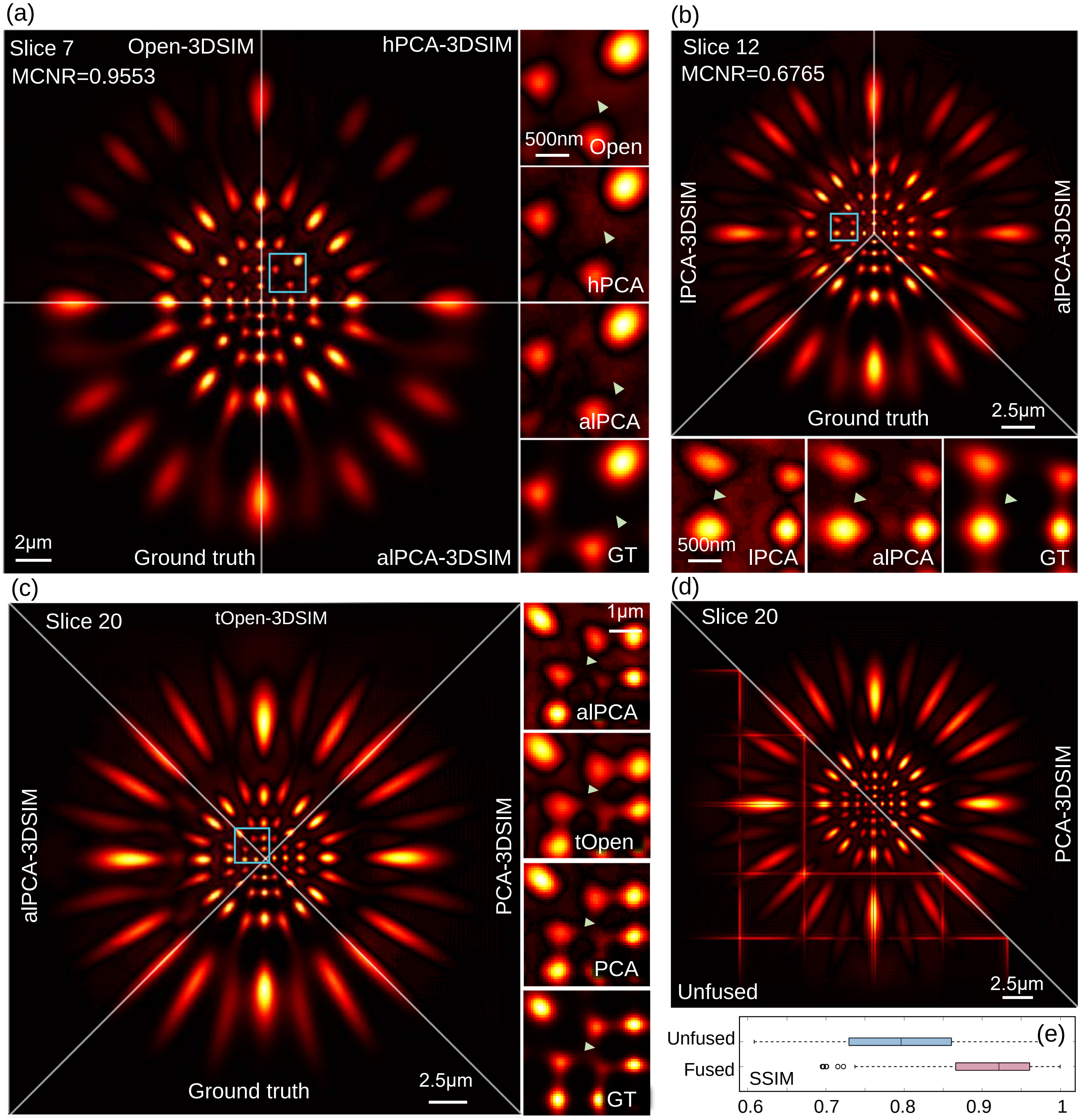

Figure S4. Comparative simulation results of different methods in the case of illumination parameters varying with volume space. (a) Comparative results of Open-3DSIM[5], hPCA-3DSIM, alPCA-3DSIM and the ground true (GT) in the regions with high MCNR values, with magnified local views to show representative detail. (b) Comparative results of lPCA-3DSIM, alPCA-3DSIM and GT in the regions where the MCNR value is less than the threshold used to judge the modulation quality, with magnified local views to show representative detail. (c) Comparative results of alPCA-3DSIM, tOpen-3DSIM, PCA-3DSIM and GT, with magnified local views to show representative detail. (d) Comparison results of PCA-3DSIM before and after image fusion. (e) SSIM between the super-resolved images obtained by PCA-3DSIM and the ground truth. The simulations were independently repeated 10 times with similar results. Colored arrows point to regions where reconstruction differences are distinct. Scale bars: 2 μm (left a); 500 nm (right a); 2.5 μm (up b, left c, d); 500 nm (down b); 1 μm (right c). Scale on z-axis: 30 layers, 0.125 μm per layer.

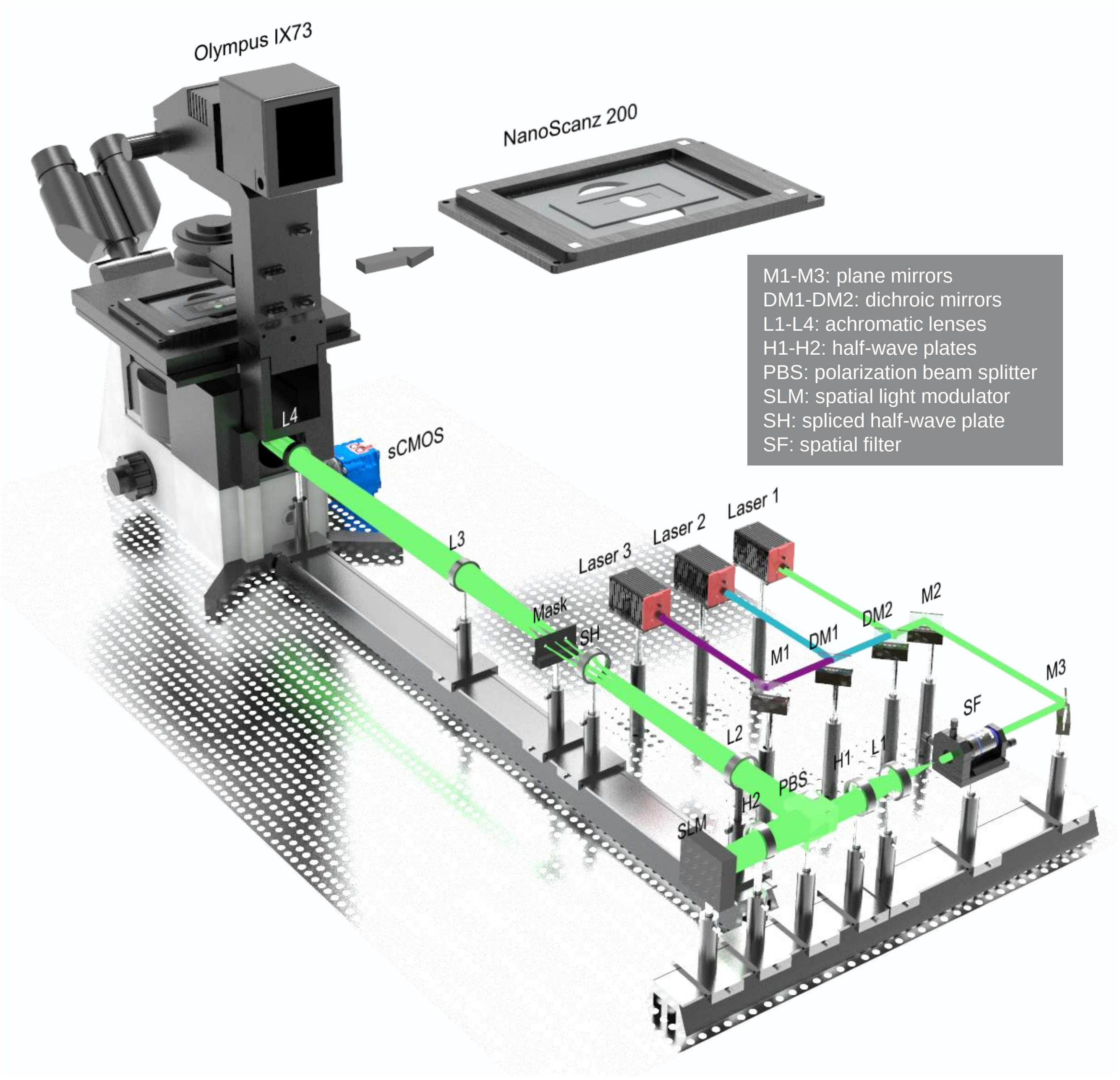

Figure S5. The self-developed multi-color 3D-SIM system. This system is implemented using an Olympus IX73 microscope with modified illumination and a nanoscale axial scanning stage (NanoScanz 200, Prior Scientific, UK).

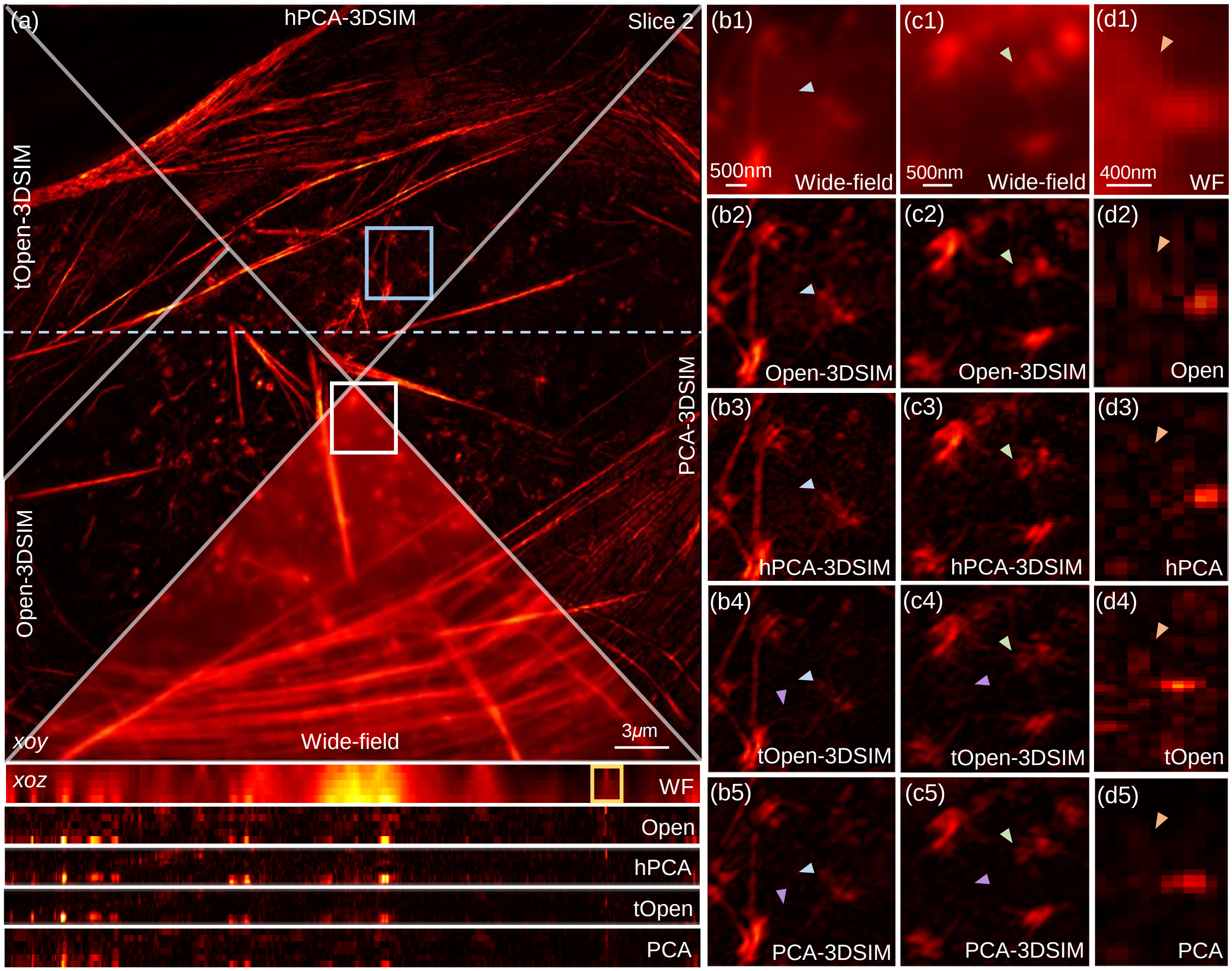

Figure S6. Comparison of the super-resolution experimental results on actin images of U2OS cells obtained by N-SIM. (a) Comparison of the wide-field image and the super-resolution images obtained by different methods (Open-3DSIM[5], hPCA-3DSIM, tOpen-3DSIM and PCA-3DSIM), where the top are lateral slices of layer 2 and the bottom are axial slices along the gray-blue dashed line. The raw SIM images come from open-source data provided in literature[5], where were captured through a 100× objective (CFI Apochromat TIRF 100× Oil, NA 1.49, Nikon, Japan) of N-SIM (Nikon, Japan). (b) Magnified wide-field image and super-resolution images from the blue-boxed regions in (a) obtained by different methods. (c) Magnified wide-field image and super-resolution images from the white-boxed regions in (a) obtained by different methods. (d) Magnified wide-field image and super-resolution images from the yellow-boxed regions in (a) obtained by different methods. The experiments were independently repeated 10 times with similar results. Colored arrows point to regions where reconstruction differences are distinct. Scale bars: 3 μm (a); 500 nm (b, c); 400 nm (d). Scale on z-axis: 12 layers, 0.125 μm per layer.

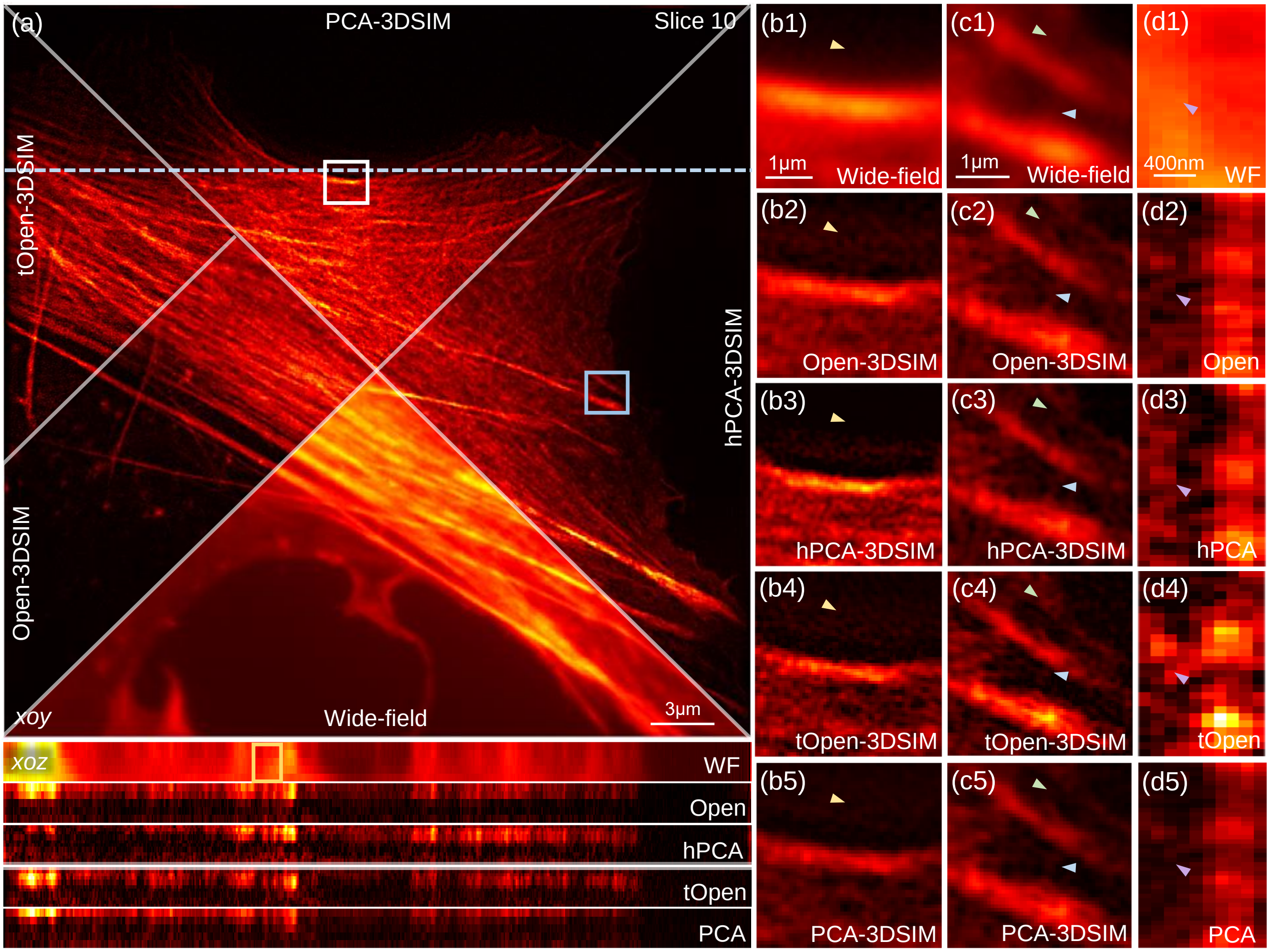

Figure S7. Comparison of the super-resolution experimental results on actin images of U2OS cells obtained by OMX-SIM at low SNRs (intensity of 2%). (a) Comparison of the wide-field image and the super-resolution images obtained by different methods (Open-3DSIM[5], hPCA-3DSIM, tOpen-3DSIM and PCA-3DSIM), where the top are lateral slices of layer 10 and the bottom are axial slices along the gray-blue dashed line. The raw SIM images come from open-source data provided in literature[5], where were captured through a 60× objective (PlanAPO 60×/1.4 Oil, Olympus, Japan) of OMX-SIM (General Electric, America). (b) Magnified wide-field image and super-resolution images from the white-boxed regions in (a) obtained by different methods. (c) Magnified wide-field image and super-resolution images from the blue-boxed regions in (a) obtained by different methods. (d) Magnified wide-field image and super-resolution images from the yellow-boxed regions in (a) obtained by different methods. The experiments were independently repeated 10 times with similar results. Colored arrows point to regions where reconstruction differences are distinct. Scale bars: 3 μm (a); 1 μm (b, c); 400 nm (d). Scale on z-axis: 10 layers, 0.125 μm per layer.

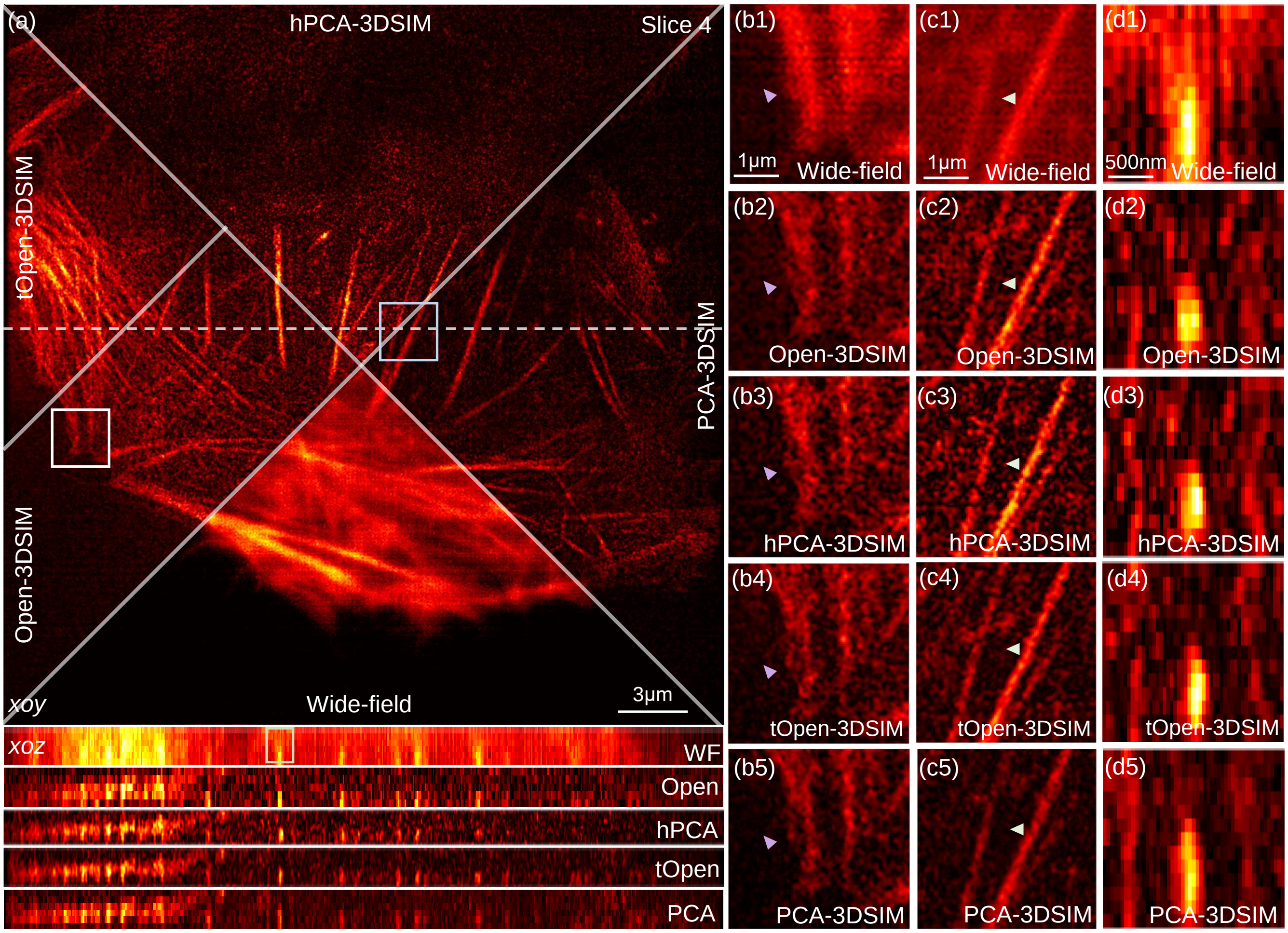

Figure S8. Comparison of the super-resolution experimental results on actin images of U2OS cells obtained by OMX-SIM at low SNRs (intensity of 2%). (a) Comparison of the wide-field image and the super-resolution images obtained by different methods (Open-3DSIM[5], hPCA-3DSIM, tOpen-3DSIM and PCA-3DSIM), where the top are lateral slices of layer 4 and the bottom are axial slices along the gray-blue dashed line. The raw SIM images come from open-source data provided in literature[5], where were captured through a 60× objective (PlanAPO 60×/1.4 Oil, Olympus, Japan) of OMX-SIM (General Electric, America). (b) Magnified wide-field image and super-resolution images from the white-boxed regions in (a) obtained by different methods. (c) Magnified wide-field image and super-resolution images from the blue-boxed regions in (a) obtained by different methods. (d) Magnified wide-field image and super-resolution images from the green-boxed regions in (a) obtained by different methods. The experiments were independently repeated 10 times with similar results. Colored arrows point to regions where reconstruction differences are distinct. Scale bars: 3 μm (a); 1 μm (b, c); 500 nm (d). Scale on z-axis: 13 layers, 0.125 μm per layer.

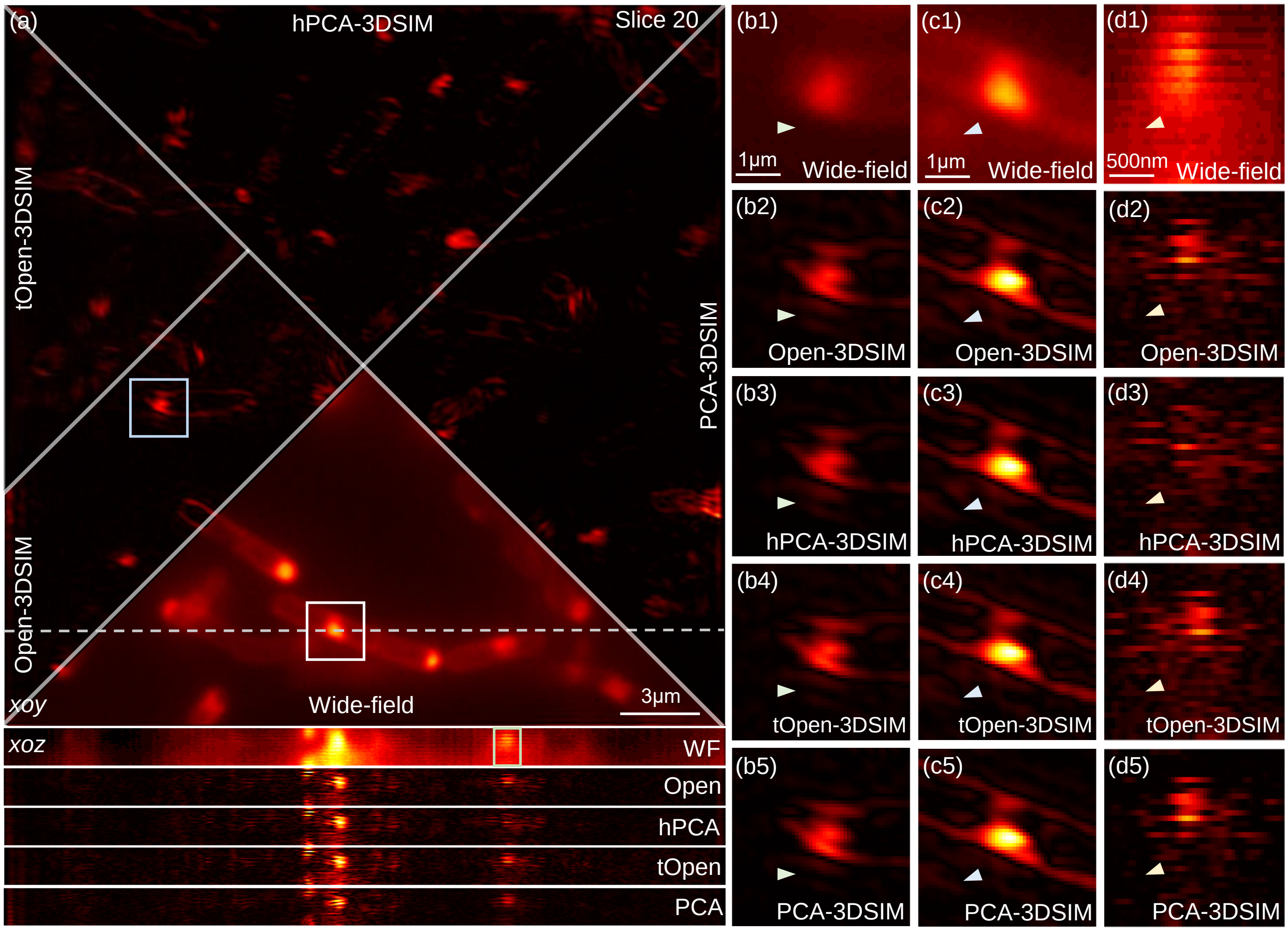

Figure S9. Comparison of the super-resolution experimental results on membrane images of live sporulating Bacillus subtilis labelled with CellBrite Fix 555. (a) Comparison of the wide-field image and the super-resolution images obtained by different methods (Open-3DSIM[5], hPCA-3DSIM, tPCA-3DSIM and PCA-3DSIM), where the top are lateral slices of layer 20 and the bottom are axial slices along the gray-blue dashed line. The raw SIM images come from open-source data provided in literature[6]. (b) Magnified wide-field image and super-resolution images from the white-boxed regions in (a) obtained by different methods. (c) Magnified wide-field image and super-resolution images from the blue-boxed regions in (a) obtained by different methods. (d) Magnified wide-field image and super-resolution images from the green-boxed regions in (a) obtained by different methods. The experiments were independently repeated 10 times with similar results. Colored arrows point to regions where reconstruction differences are distinct. Scale bars: 3 μm (a); 1 μm (b, c); 500 nm (d). Scale on z-axis: 32 layers, 0.2 μm per layer.

Table S2. The execution time of each key step when running the open-source code using the original data in Fig. S7 as an example.

Algorithm step	Time consumption/s	Repeat count times	Methods of acceleration
MCNR acquisition for individual blocks	0.072002 ± 0.000760	7×7	GPU parallel acceleration
PCA-based parameter estimation for individual tiles	0.032045 ± 0.000616	7×7×10	Multi-threaded parallel acceleration
Image reconstruction for individual blocks	0.556789 ± 0.006189	7×7	GPU parallel acceleration combined with multi-threaded parallel acceleration
Image fusion	0.842313 ± 0.011328	/	GPU parallel acceleration combined with multi-threaded parallel acceleration
Total	637.170462 ± 4.353022	/	/

Supporting Information S6. Supplementary code for PCA-3DSIM

We provide the open-source code of PCA-3DSIM, which is available at: https://figshare.com/s/93ef85f7b6f0a40f75f2. In this section, we will introduce the configuration environment and the file structure of open-source information, as well as the operation guide.

1. Configuration environment

The open-source software is modified on the basis of the code of Open-3DSIM[5]. It is developed on the MATLAB platform (version R2018a or later), and integrates open-source toolkits, including Bio-Formats and Dip_Image, to facilitate the import of `*.nd2`, `*.dv`, `*.tif`, and `*.tiff` image formats and to enable 3D frequency-domain transformations. All required dependencies are provided within the `lib` directory for ease of configuration and reproducibility.

2. File structure

The raw input images should be placed in the `input` directory. The program automatically identifies and processes files in `*.nd2`, `*.tif`, `*.tiff`, and `*.dv` formats. The reconstructed results of PCA-3DSIM are generated and saved in the `output` directory. Example files include:

- `PCA-3DSIM/input/cos_7.tif`: an example input file.
- `PCA-3DSIM/output/PCA_3DSIM`: reconstruction results of PCA-3DSIM corresponding to the input.
- `PCA-3DSIM/output/WF`: wide-field images corresponding to the input.
- `PCA-3DSIM/output/WF2`: wide-field images after pixel upsampling.
- `PCA-3DSIM/output/Open_3DSIM`: reconstruction results of Open-3DSIM for comparison.

3. Operation Steps

A. Place the original 3D images in the `input` folder. Supported formats include `*.tif`, `*.tiff`, `*.nd2`, and `*.dv`, which are commonly generated by OMX, N-SIM, or custom-built 3D-SIM systems.

B. Open `PCA_3DSIM.m` in MATLAB and adjust the acquisition parameters according to the specific characteristics of the input data.

C. Execute `PCA_3DSIM.m`. The wide-field image (`WF.tif`, `WF2.tif`) and the corresponding PCA-3DSIM reconstruction (`PCA_3DSIM.tif`) will be automatically saved to the `output` folder.

D. For result visualization and detailed comparison, the `show_result()` function can be executed. This function is configured for the provided example dataset and requires modification for other input data.

Supporting Information S7. Supplementary Videos

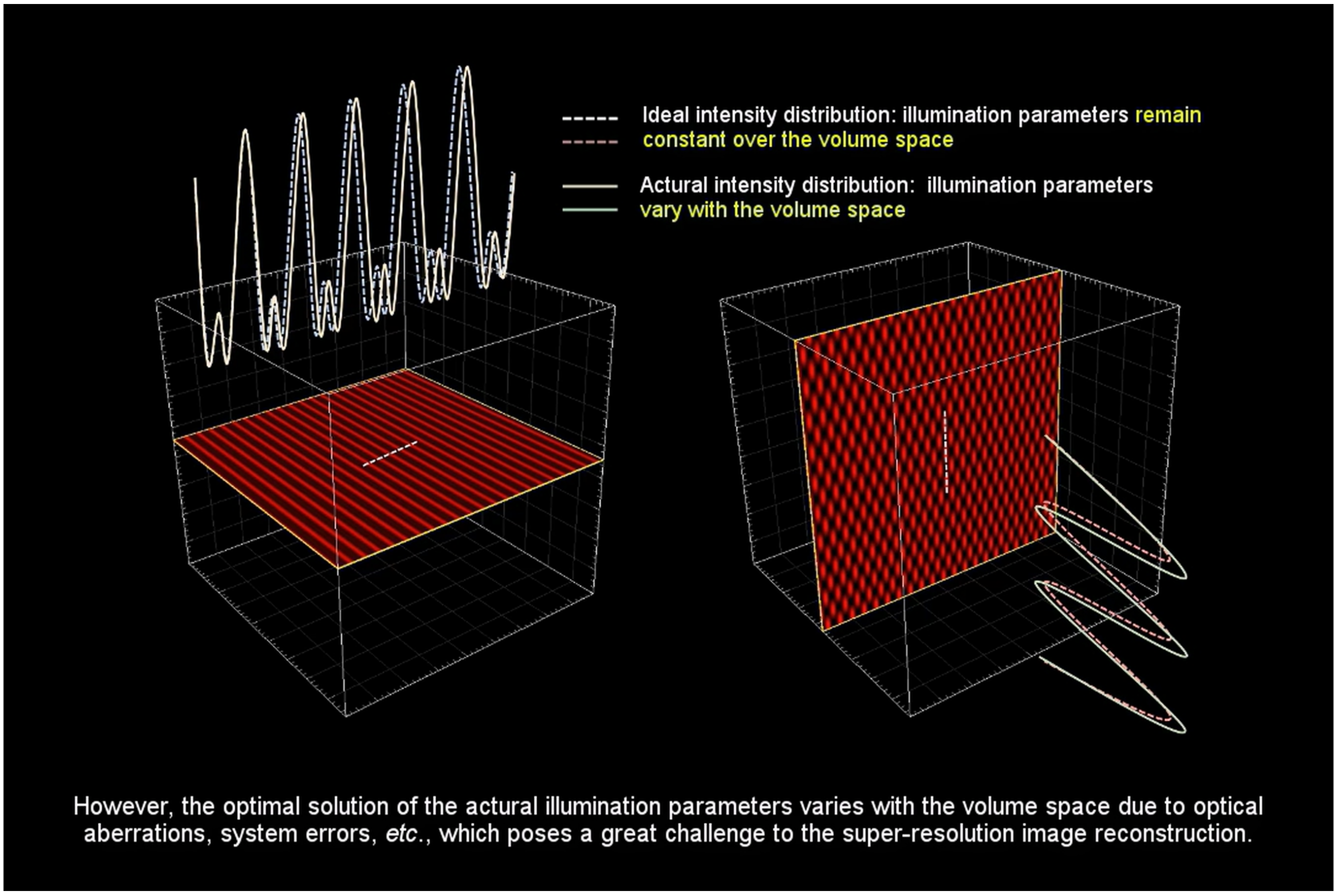

Figure S10. Movie S1: Illumination modulation in 3DSIM and the significance of illumination parameter estimation for super-resolution reconstruction.

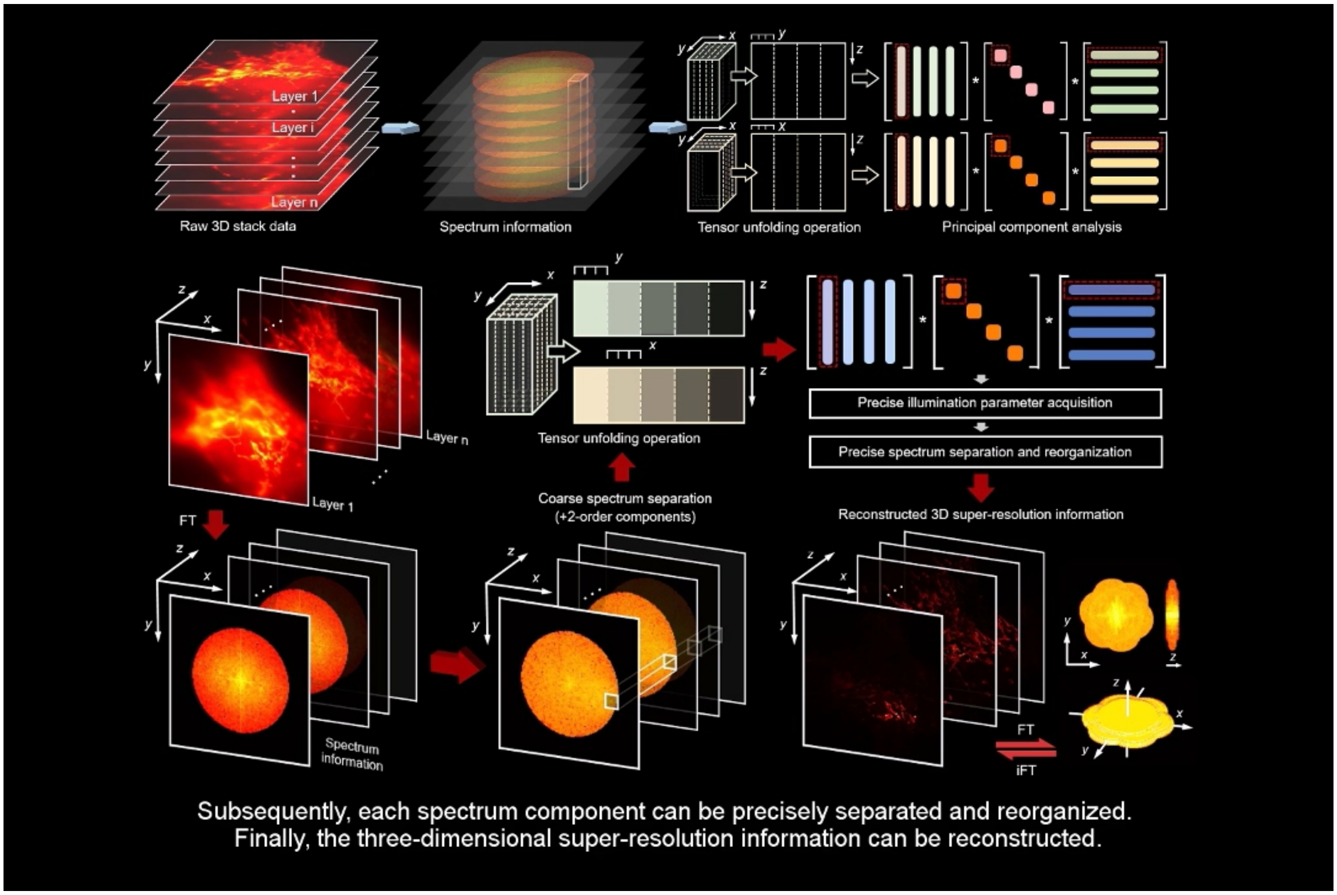

Figure S11. Movie S2: The principle of three-dimensional structured illumination microscopy based on principal component analysis (PCA-3DSIM) without the adaptive tiled-block strategy.

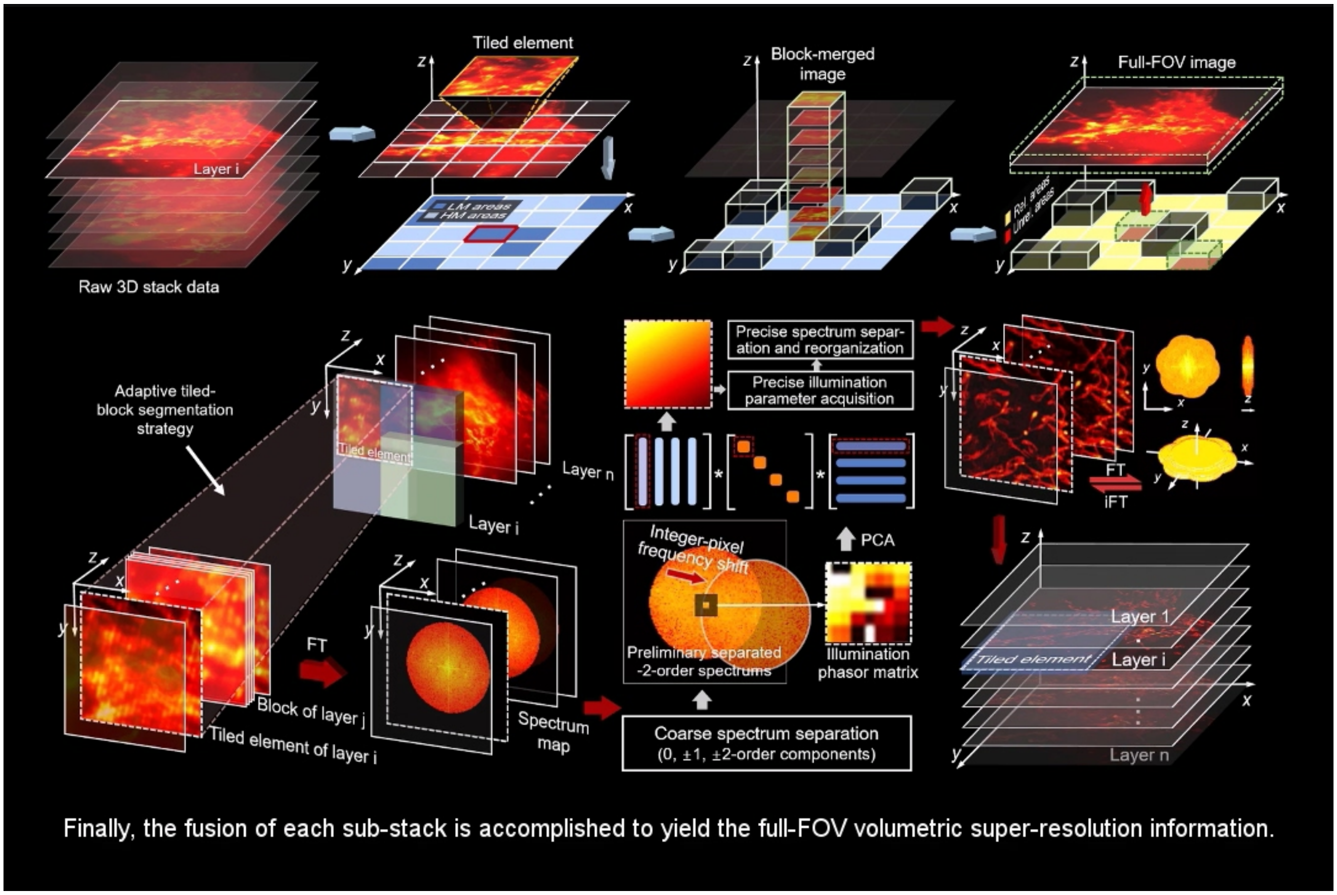

Figure S12. Movie S3: The principle of three-dimensional structured illumination microscopy based on principal component analysis (PCA-3DSIM).

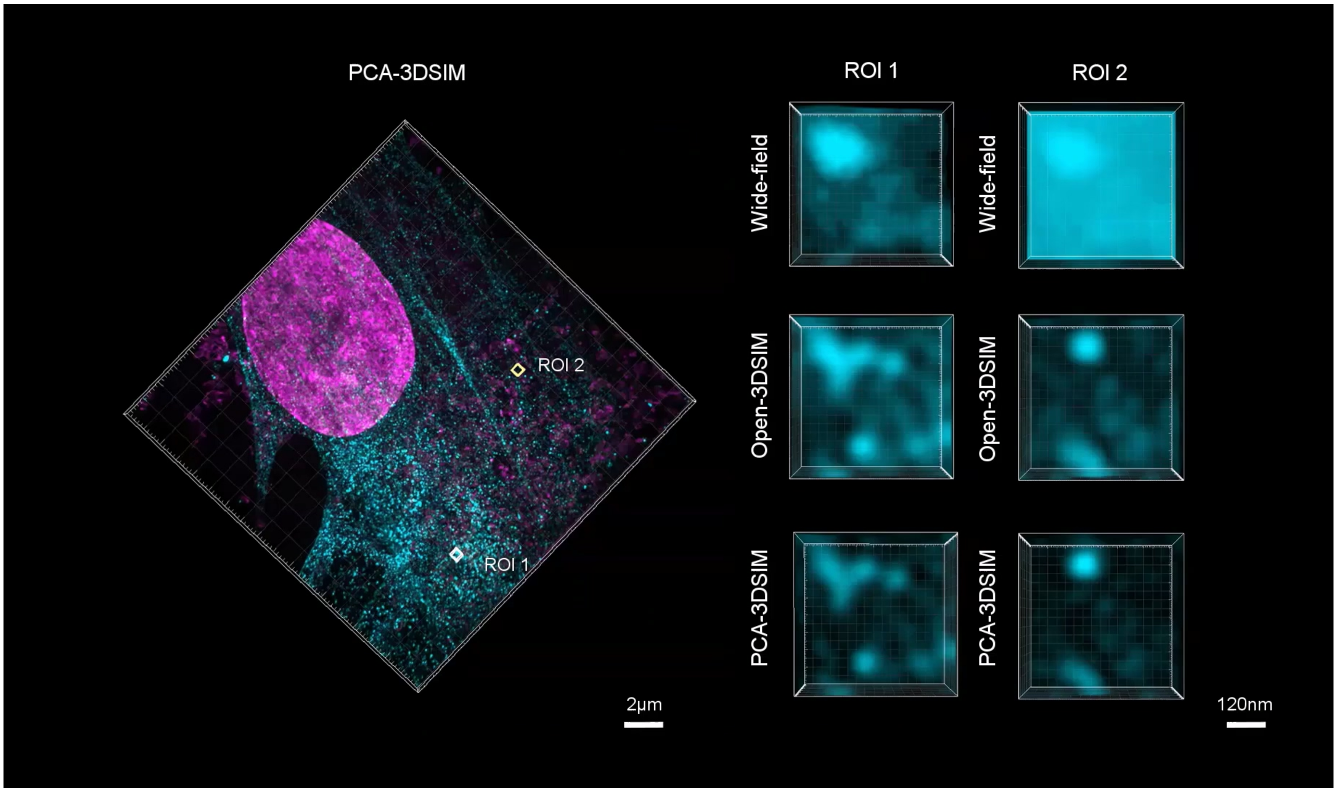

Figure S13. Movie S4: The comparison between the 3D volumetric reconstruction results of HeLa cells obtained using PCA-3DSIM and Open-3DSIM, respectively.

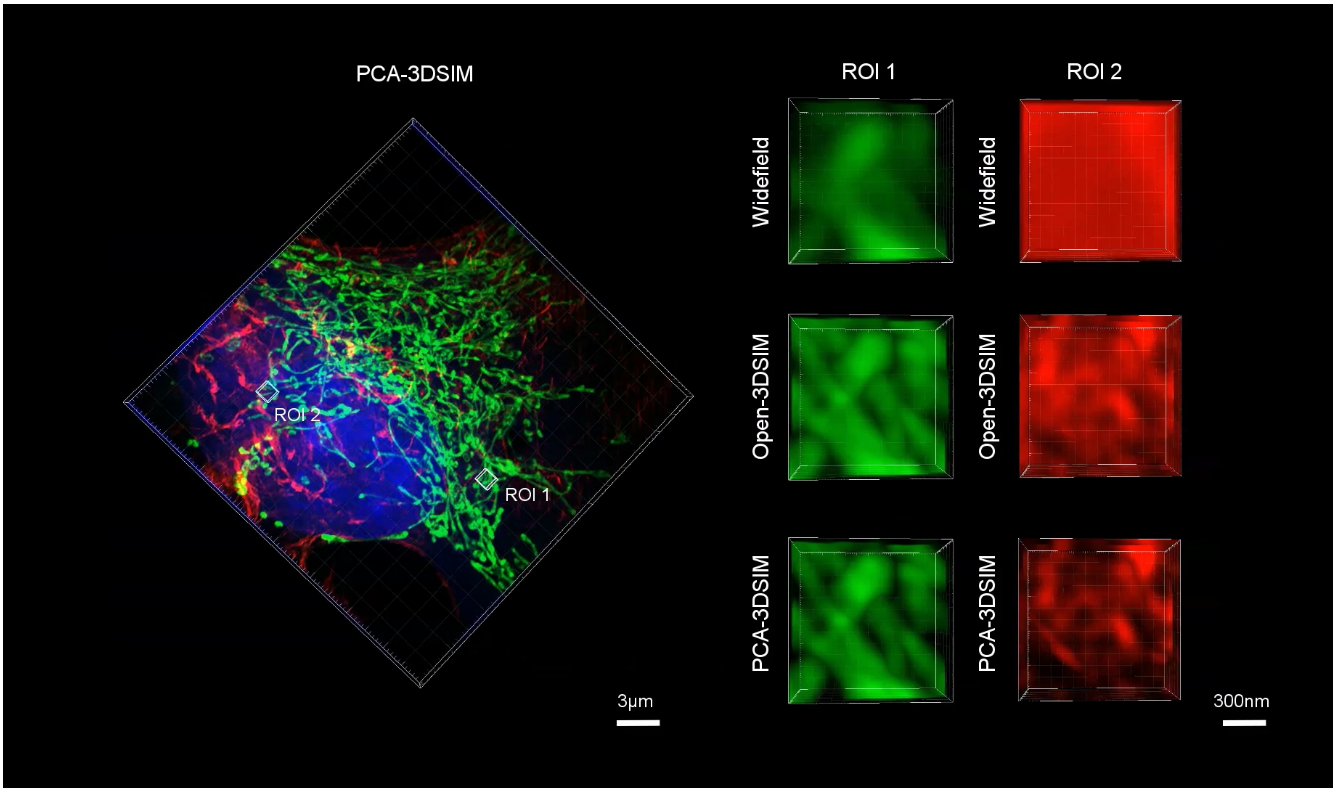

Figure S14. Movie S5: The comparison between the 3D volumetric reconstruction results of COS-7 cells obtained using PCA-3DSIM and Open-3DSIM, respectively.

References

1. Gustafsson, M. G. Surpassing the lateral resolution limit by a factor of two using structured illumination microscopy. *J. Microsc.* **198**, 82–87 (2000).
2. Gustafsson, M. G. *et al.* Three-dimensional resolution doubling in wide-field fluorescence microscopy by structured illumination. *Biophys. J.* **94**, 4957–4970 (2008).
3. Fiolka, R., Shao, L., Rego, E. H., Davidson, M. W. & Gustafsson, M. G. Time-lapse two-color 3d imaging of live cells with doubled resolution using structured illumination. *Proc. Natl. Acad. Sci.* **109**, 5311–5315 (2012).
4. Demmerle, J. *et al.* Strategic and practical guidelines for successful structured illumination microscopy. *Nat. Protoc.* **12**, 988–1010 (2017).
5. Cao, R. *et al.* Open-3dsim: an open-source three-dimensional structured illumination microscopy reconstruction platform. *Nat. Methods* **20**, 1183–1186 (2023).
6. Li, X. *et al.* Three-dimensional structured illumination microscopy with enhanced axial resolution. *Nat. Biotechnol.* **41**, 1307–1319 (2023).
7. Qian, J. *et al.* Structured illumination microscopy based on principal component analysis. *eLight* **3**, 4 (2023).
8. Shi, Q., Lu, H. & Cheung, Y.-m. Tensor rank estimation and completion via cp-based nuclear norm. In *Proceedings of the 2017 ACM on Conference on Information and Knowledge Management*, 949–958 (2017).
9. Hoge, W. S. & Westin, C.-F. Identification of translational displacements between n-dimensional data sets using the high-order svd and phase correlation. *IEEE Transactions on Image Process.* **14**, 884–889 (2005).
10. De Lathauwer, L., De Moor, B. & Vandewalle, J. A multilinear singular value decomposition. *SIAM journal on Matrix Analysis Appl.* **21**, 1253–1278 (2000).
11. Diniz, P. S., Da Silva, E. A. & Netto, S. L. *Digital signal processing: system analysis and design* (Cambridge University Press, 2010).
12. Wen, G. *et al.* High-fidelity structured illumination microscopy by point-spread-function engineering. *Light. Sci. & Appl.* **10**, 70 (2021).
